# A host-microbiome interaction mediates the opposing effects of omega-6 and omega-3 fatty acids on metabolic endotoxemia

**DOI:** 10.1038/srep11276

**Published:** 2015-06-11

**Authors:** Kanakaraju Kaliannan, Bin Wang, Xiang-Yong Li, Kui-Jin Kim, Jing X. Kang

**Affiliations:** 1 Laboratory for Lipid Medicine and Technology, Department of Medicine, Massachusetts General Hospital and Harvard Medical School, Boston, MA 02129, USA

## Abstract

Metabolic endotoxemia, commonly derived from gut dysbiosis, is a primary cause of chronic low grade inflammation that underlies many chronic diseases. Here we show that mice fed a diet high in omega-6 fatty acids exhibit higher levels of metabolic endotoxemia and systemic low-grade inflammation, while transgenic conversion of tissue omega-6 to omega-3 fatty acids dramatically reduces endotoxemic and inflammatory status. These opposing effects of tissue omega-6 and omega-3 fatty acids can be eliminated by antibiotic treatment and animal co-housing, suggesting the involvement of the gut microbiota. Analysis of gut microbiota and fecal transfer revealed that elevated tissue omega-3 fatty acids enhance intestinal production and secretion of intestinal alkaline phosphatase (IAP), which induces changes in the gut bacteria composition resulting in decreased lipopolysaccharide production and gut permeability, and ultimately, reduced metabolic endotoxemia and inflammation. Our findings uncover an interaction between host tissue fatty acid composition and gut microbiota as a novel mechanism for the anti-inflammatory effect of omega-3 fatty acids. Given the excess of omega-6 and deficiency of omega-3 in the modern Western diet, the differential effects of tissue omega-6 and omega-3 fatty acids on gut microbiota and metabolic endotoxemia provide insight into the etiology and management of today’s health epidemics.

Chronic low-grade inflammation is now considered to be a critical pathological factor underlying many modern chronic diseases, including diabetes, cardiovascular disease, cancer, and neurodegenerative diseases, and is associated with aging^1^,^2^,^3^. Chronic low-grade inflammation is characterized by elevated circulating levels of inflammatory cytokines, such as tumor necrosis factor-alpha (TNF-α), interleukin (IL)-1, and IL-6^1^,^2^,^3^. A primary cause of chronic low-grade inflammation is metabolic endotoxemia^4^, which presents as a gradual increase in plasma endotoxins, particularly lipopolysaccharides (LPS). The binding of LPS with Toll-like receptor-4 (TLR4) and subsequent activation of NLRP3 inflammasome leads to the increased expression of inflammatory cytokines^5^,^6^. Understanding the factors that modulate the development of metabolic endotoxemia is important for the management of chronic disease. Metabolic endotoxemia can often result from gut dysbiosis, such as an increase in LPS-producing bacteria (e.g. *Enterobactericeae*) and/or a decrease in LPS-suppressing bacteria (those which can lower the numbers of LPS-producing bacteria, such as *Bifidobacterium*), as well as intestinal barrier dysfunction^7^,^8^,^9^. Diet is known to be an important modulating factor of metabolic endotoxemia and chronic low-grade inflammation^8^,^9^,^10^,^11^; for example, a high-saturated fat diet can increase LPS levels and induce endotoxemia^10^,^11^. Clearly, any factor that can alter the gut microbiota could play a role in regulating metabolic endotoxemia and chronic low-grade inflammation. Identifying the dietary components that can optimize the gut microbiota will be crucial for the prevention and treatment of chronic disease.

It is well recognized that long-chain omega-6 (n-6) and omega-3 (n-3) polyunsaturated fatty acids (PUFA) play important and opposing roles in the modulation of inflammation. Generally, n-6 PUFA promote inflammation, whereas n-3 PUFA have anti-inflammatory properties^12^,^13^. Since n-6 and n-3 long-chain PUFA compete for the same enzymes for their synthesis and metabolism, their ratio in body tissues determines the profile of lipid mediators involved in the inflammatory response. Over the past decades, the n-6/n-3 PUFA ratio has undergone a dramatic shift from the human evolutionary ratio of ~1:1 to the modern dietary ratio ranging from 10:1 to 50:1, due to the increased intake of foods rich in n-6 PUFA and deficient in n-3 PUFA^14^,^15^,^16^. This shift in the n-6/n-3 PUFA ratio is thought to contribute to today’s prevalence of chronic disease^17^,^18^. In fact, much research has demonstrated the beneficial effects of n-3 PUFA supplementation on various chronic diseases, which have been attributed to their anti-inflammatory properties^12^,^17^,^18^,^19^. Many lines of evidence, from animals to humans, have shown that elevated tissue n-3 PUFA levels can suppress the production of inflammatory cytokines, particularly TNF-α, IL-1β, and IL-6^20^. A number of mechanisms have been proposed for the opposing effects of n-6/n-3 PUFA, including (a) competing with n-6 PUFA for metabolism and reducing the production of n-6 PUFA-derived inflammatory eicosanoids; (b) down-regulating the key enzymes (e.g. COX-2) that synthesize lipid mediators; (c) directly acting on transcriptional factors (e.g. NF-κB) to inhibit the expression of inflammatory cytokines; and (d) producing potent inflammation-resolving compounds (e.g. resolvins and protectins)^20^. However, whether the opposing effects of n-6 and n-3 PUFA on inflammation are associated with their effects on gut microbiota remains unknown.

Given the critical role of metabolic endotoxemia in the development of chronic low-grade inflammation, we hypothesize that the opposing effects of tissue n-6 and n-3 PUFA on inflammation are due to their differential regulation of metabolic endotoxemia through altering the gut microbiota. To examine the impact of the tissue n-6/n-3 PUFA composition on gut microbiota-associated endotoxemia, here we utilized the fat-1 transgenic mouse model, which expresses the *Caenorhabditis elegans* fat-1 gene and is capable of producing n-3 PUFA from n-6 PUFA without the need for dietary n-3 PUFA supplementation^22^. This model allows us to use a single diet to generate two distinct tissue fatty acid profiles – one with a high n-6/n-3 PUFA ratio similar to that of the Western diet (>10:1), and the other with a balanced n-6/n-3 PUFA ratio (~1:1) – thereby eliminating the typical confounding factors of diet. Our findings reveal an interaction between the host tissue fatty acid composition and the gut microbiota and provide a novel mechanism for the opposing effects of n-6 and n-3 PUFA on inflammation through the modulation of metabolic endotoxemia.

## Results

### Transgenic conversion of tissue n-6 to n-3 PUFA reduces metabolic endotoxemia and systemic low-grade inflammation

**Figure 1:**
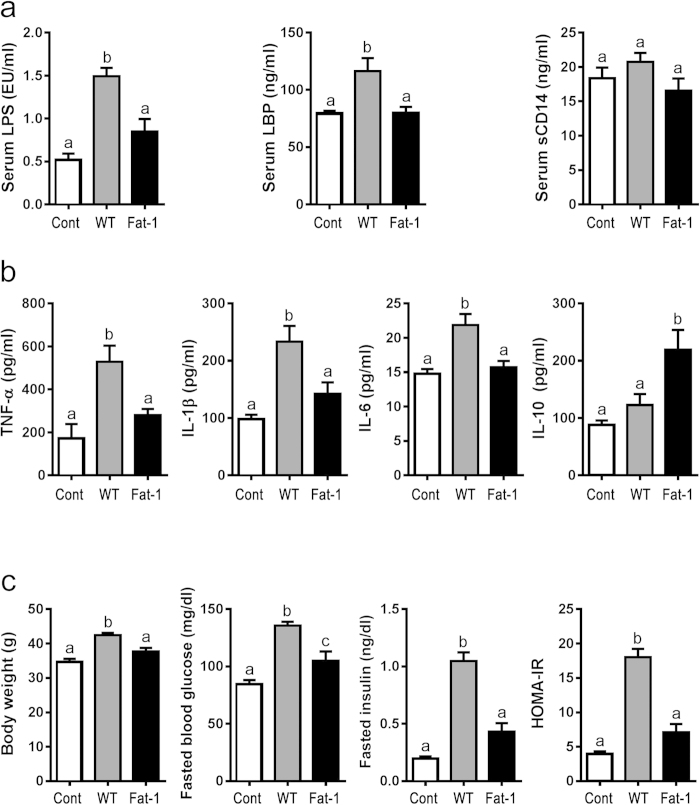
Endogenous conversion of omega-6 to omega-3 fatty acids reduces metabolic endotoxemia and systemic low grade inflammation. C57BL/6 male WT (n = 10) and fat-1 transgenic mice (n = 10) were maintained on the same diet high in n-6 PUFA since weaning for 8 months. A group of age-matched male WT mice fed with a chow diet (n = 10) was used as control. Blood samples were collected from all the mice at the same time and subjected to various analyses. **(a)** Parameters of metabolic endotoxemia (LPS, LBP and sCD14); **(b)** Serum levels of inflammation-related cytokines (TNF-α, IL-1β, IL-6, and IL-10) and **(c)** Markers of metabolic syndrome (body weight, fasting blood glucose, fasting insulin, and HOMA-IR). Data are expressed as mean ± SE. Data with different superscript letters are significantly different (P < 0.05) according to one-way ANOVA with Tukey test.

The increased dietary intake of n-6 PUFA and decreased intake of n-3 PUFA has coincided with the growing incidence of chronic diseases. To investigate whether this shift in the essential fatty acid composition affects metabolic endotoxemia, a risk factor for chronic disease, we utilized the unique transgenic fat-1 mouse model to examine markers for endotoxemia and chronic low-grade inflammation in animals with varying tissue essential fatty acid content. As shown in Fig. 1a, 8-month-old WT mice fed a diet rich in n-6 PUFA, with an intestinal tissue n-6/n-3 PUFA ratio of ~25:1 (Supplementary Fig. S1), exhibited dramatically elevated serum levels of metabolic endotoxemia markers, namely LPS and LPS-binding protein (LBP), compared to the control group. Interestingly, endogenous conversion of n-6 to n-3 PUFA in the fat-1 mice, fed on the same diet but with a tissue n-6/n-3 PUFA ratio of ~4:1 (Supplementary Fig. S1), significantly reduced the levels of these markers (Fig. 1a). Accordingly, the increased levels of serum inflammatory cytokines (TNF-α, IL-1β, and IL-6) induced by high n-6 PUFA tissue content were decreased by conversion of n-6 to n-3 PUFA, while levels of the anti-inflammatory cytokine IL-10 were significantly increased (Fig. 1b). Furthermore, typical markers of metabolic syndrome (body weight, fasting blood glucose, fasting insulin, and HOMA-IR) were similarly altered by changing the tissue essential fatty acid composition (Fig. 1c), although fasting blood glucose was not completely reversed in fat-1 mice. Notably, we observed even more significant differences in aged, 20-month-old mice between the WT and fat-1 groups in the markers of metabolic endotoxemia, inflammation, and metabolic syndrome, as well as downstream factors of the pathways of LPS-TLR4 (LBP, CD14, TLR4 and NFκB-p65) and LPS-NLRP3 inflammasome (NLRP3, ASC, Caspase-1 and Pannexin) (Supplementary Fig. S2). These results demonstrate, in an experimental system free of the confounding factors of diet, that tissue n-6 and n-3 PUFA content have opposing effects on metabolic endotoxemia and inflammation.

### Gut microbiota mediate the differential effects of tissue n-6 and n-3 PUFA content on metabolic endotoxemia

**Figure 2:**
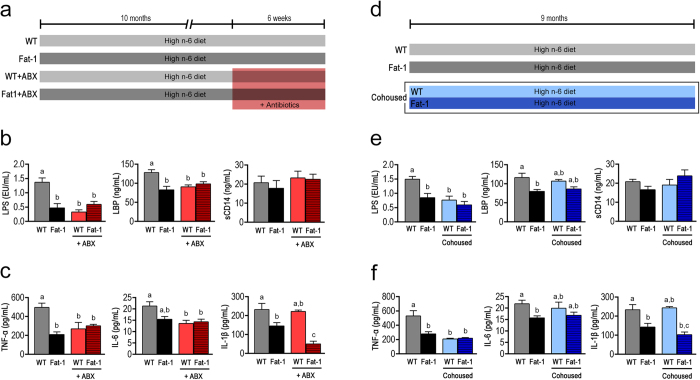
Antibiotic treatment and co-housing alter the effects of tissue omega-6 and omega-3 PUFA status on metabolic endotoxemia and inflammation. (**a**) Schema showing the animal groups and treatments. Separately housed 10-month-old male WT (n = 10) and fat-1 (n = 10) were maintained on the same high n-6 diet, and half in each group received a broad spectrum antibiotic cocktail (ABX) consisting of ampicillin (1 g/L), vancomycin (500 mg/L), neomycin sulfate (1 g/L) (added to drinking water) and metronidazole (100 mg/kg) (orally gavaged every 12 h) for 6 weeks. After treatment, the differences between WT and fat-1 mice in many parameters related to endotoxemia (**b**) and inflammation (**c**) were eliminated. For the co-housing experiment, 9-month-old fat-1 (n = 4) and WT littermates (n = 4) were co-housed in two cages (2 mice from each genotype/cage) and fed an identical high n-6 diet after weaning (**d**). There was no difference between the co-housed WT and fat-1 mice in many parameters related to endotoxemia (**e**) and inflammation (**f**), compared to the separately housed WT and fat-1 mice. Data are expressed as mean ± SE. Data with different superscript letters are significantly different (P < 0.05) according to one-way ANOVA with Tukey test.

Given that metabolic endotoxemia is commonly derived from gut dysbiosis, we next determined whether gut microbiota are necessary for the effects of tissue essential fatty acid composition on metabolic endotoxemia using antibiotic treatment and co-housing experiments. We used broad spectrum antibiotics (ampicillin, metronidazole, neomycin and vancomycin) to create groups of macroscopically germ-free WT (WT+ABX) and fat-1 (Fat1+ABX) mice, which were both fed a high n-6 PUFA diet (Fig. 2a). Following treatment with antibiotics, the differences observed between the WT and fat-1 mice in markers of metabolic endotoxemia and inflammation were eliminated with the exception of IL-1β and serum TG, as the WT+ABX group exhibited an improved metabolic profile similar to that of the Fat1+ABX mice (Fig. 2b,c and Supplementary Fig. S3). These findings suggest that the gut microbiota may mediate the differential effects of tissue n-6 and n-3 PUFA on metabolic endotoxemia; more specifically, mice with a high tissue n-6/n-3 PUFA ratio appear to harbor pro-endotoxic bacteria.

Co-housing of mice permits horizontal transmission of fecal material, which has been shown to facilitate transfer of disease among mice^23^,^24^. Following co-housing of WT mice with fat-1 littermates for 9 months on the same high n-6 diet (Fig. 2d), we found that the co-housed WT mice closely resembled the fat-1 mice in the majority of parameters related to metabolic endotoxemia and inflammation except for IL-1β and serum TG (Fig. 2e,f and Supplementary Fig. S4). These results suggest that fat-1 mice produce gut microbiota-modifying factors that are transmissible to WT mice through long-term co-housing and can alter their gut microbiota accordingly.

**Figure 3:**
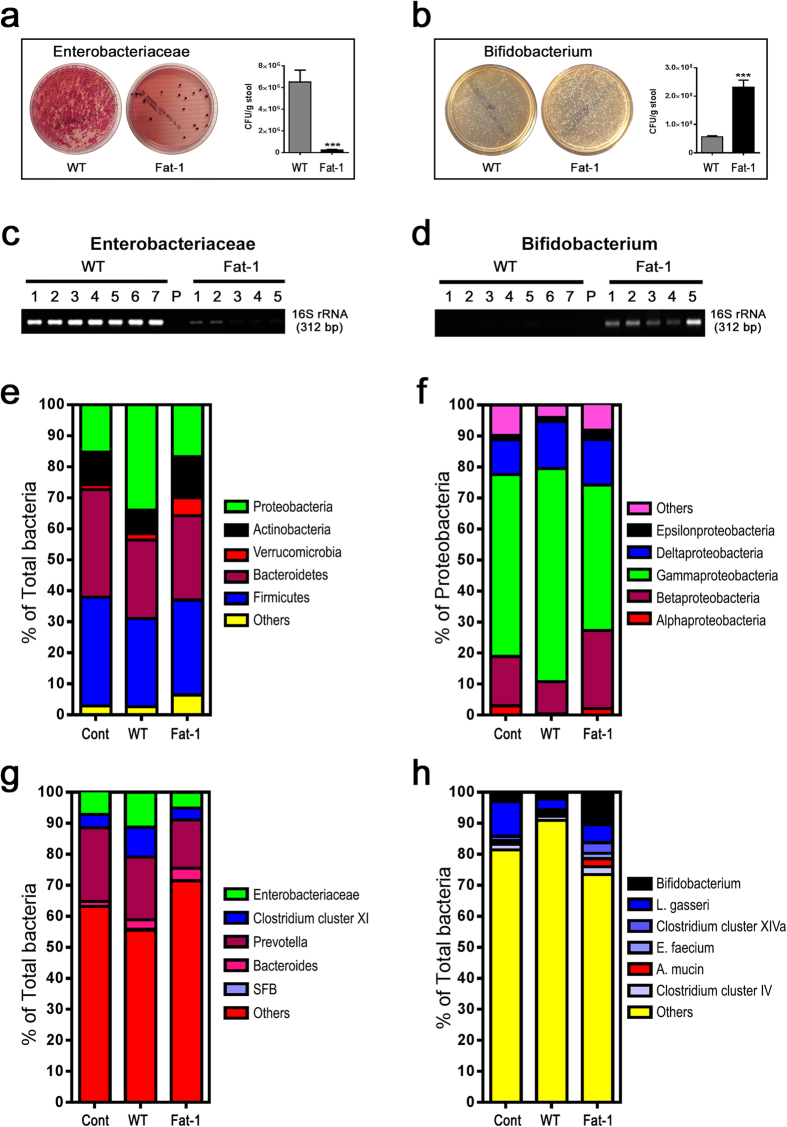
Transgenic conversion of tissue omega-6 to omega-3 PUFA alters gut microbiota. Eight-month-old C57BL/6 male WT (n = 10) and fat-1 transgenic mice (n = 10) were maintained on the same diet high in n-6 PUFA since weaning. A group of age-matched male WT mice fed with a chow diet (n = 10) was used as control. Stool samples from all groups of mice were subjected to quantification of microbiota by qPCR and bacterial stool culture. (**a & b**) Representative culture plate photos showing the difference between WT and fat-1 mice in the growth of LPS-producing gram negative *Enterobacteriaceae* and LPS-suppressing *Bifidobacterium* species. (**c & d**) Gel photos showing quantitative PCR products for *Enterobacteriaceae* and *Bifidobacterium* amplified from equal amounts of stool DNA from WT and fat-1 mice. (**e**) Relative abundance of the 5 most dominant phyla in the stool between groups. (**f**) Relative abundance of major classes of LPS-producing members of the *Proteobacteria* phylum between groups. (**g**) Relative abundance of major groups of LPS-producing and/or pro-inflammatory bacteria at the sub-phylum level. (**h**) Relative abundance of major groups of LPS-suppressing and/or anti-inflammatory bacteria at the sub-phylum level. Specific primers used are shown in Supplementary Table 3. For panels a & b, stool culture results were shown as colony forming unit per gram stool (CFU/g stool) and expressed as mean ± SE, and significance was determined by unpaired two-tailed student T-test. *** *P *< 0.001.

The observations from the antibiotic treatment and cohousing experiments led us to examine the gut microbiota profiles between the WT and fat-1 mice, with a focus on bacteria linked to LPS production, gut inflammation and metabolic syndrome (Supplementary Table S1). Using both bacterial culture and quantitative PCR to identify and quantify the bacteria in stool, we found that the fat-1 mice exhibited significantly lowered or undetectable quantities of LPS-producing and/or pro-inflammatory bacterial groups, including the phylum *Proteobacteria* and its members (*Enterobacteriaceae, Escherichia coli, gamma-* and *delta-proteobacteria*), *Prevotella, Fusobacterium*, *Clostridium* cluster XI, and Segmented Filamentous Bacteria (SFB), compared to their WT littermates (Fig. 3 and Supplementary Fig. S5). In contrast, the levels of LPS-suppressing and/or anti-inflammatory bacterial groups, such as *Bifidobacterium*, *Akkermansia muciniphila*, *Lactobacillus* (primarily *L. gasseri*)*, Clostridium* clusters IV and XIVa, and *Enterococcus faecium*, were markedly higher in the fat-1 mice compared to their WT littermates (Fig. 3 and Supplementary Fig. S5). Notably, the bacterial overgrowth in the WT group was mainly due to the increased proportion of LPS-producing members of the phylum *Proteobacteria* (Fig. 3 and Supplementary Fig. S5). Intestinal permeability was also lower in the fat-1 mice than in the WT mice (Supplementary Fig. S6a–c). As anticipated, the overall stool microbiota profile of the co-housed WT mice was similar to those of the fat-1 groups, particularly the abundance of stool bacteria favoring or suppressing metabolic endotoxemia (Supplementary Figs. S7,S8). These findings indicate that the tissue n-6/n-3 PUFA ratio is a determinant of the gut microbiota profile: a high tissue n-6/n-3 PUFA ratio can increase the proportions of LPS-producing and/or pro-inflammatory bacteria and decrease those of LPS-suppressing and/or anti-inflammatory bacteria, while reducing the tissue n-6/n-3 PUFA ratio has the opposite effect.

### IAP is a gut microbiota-modifying factor regulated by the tissue essential fatty acid composition

**Figure 4:**
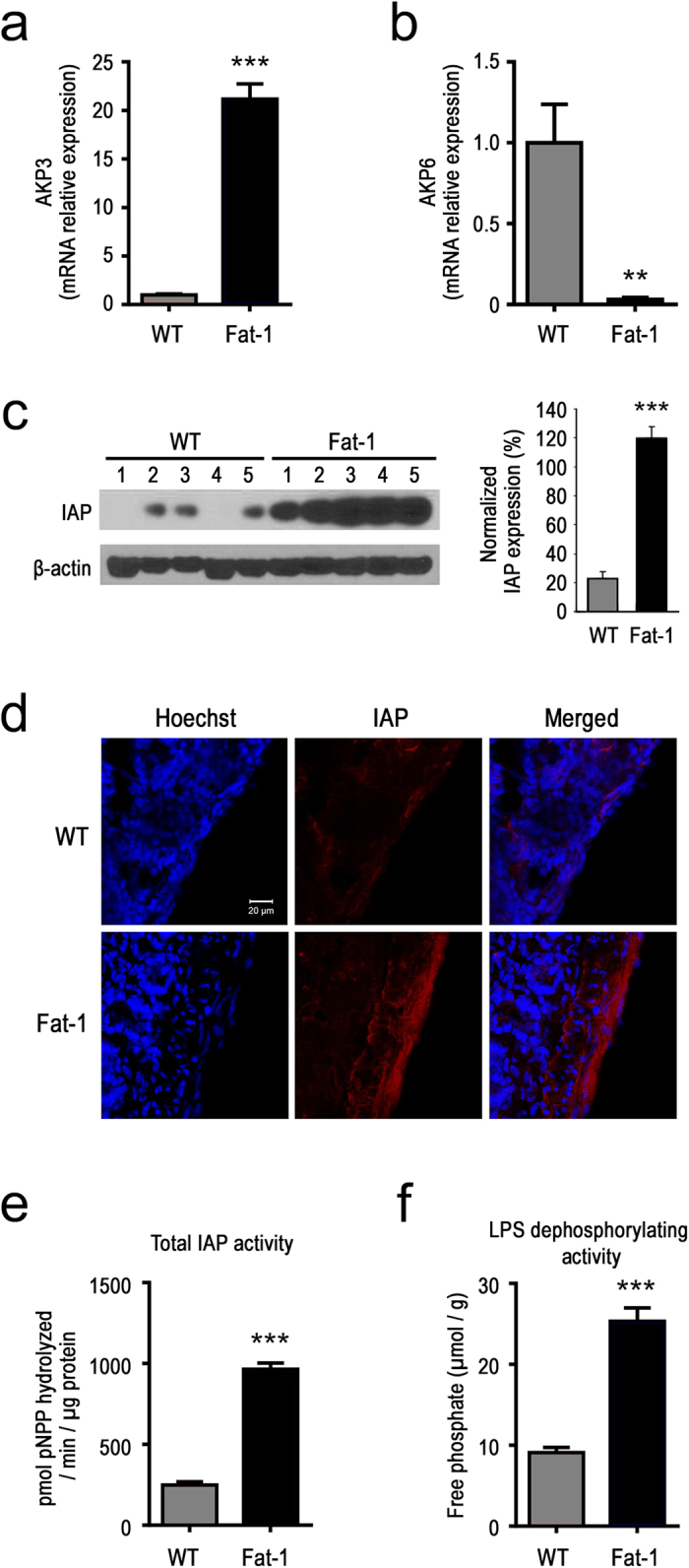
Elevated tissue omega-3 PUFA increases the expression and activity of intestinal alkaline phosphatase (IAP). (**a & b**) Relative mRNA expression of two major IAP isozymes (AKP 3 and AKP 6) in the intestine of 20 month old WT (n = 4) and fat-1 mice (n = 5); **(c)** Western blot analysis showing the protein levels of IAP in WT (n = 5) and fat-1 mice (n = 5); **(d)** Representative immunofluorescent images showing the difference in IAP expression in of small intestine between WT and fat-1 mice; **(e)** Total IAP activity and **(f)** LPS-dephosphorylating activity of small intestinal tissue from WT (n = 4) and fat-1 (n = 5) mice. Data are expressed as mean ± SE. Significance was determined by unpaired two-tailed student T-test. *P < 0.05; ** *P *< 0.01; *** *P *< 0.001.

**Figure 5:**
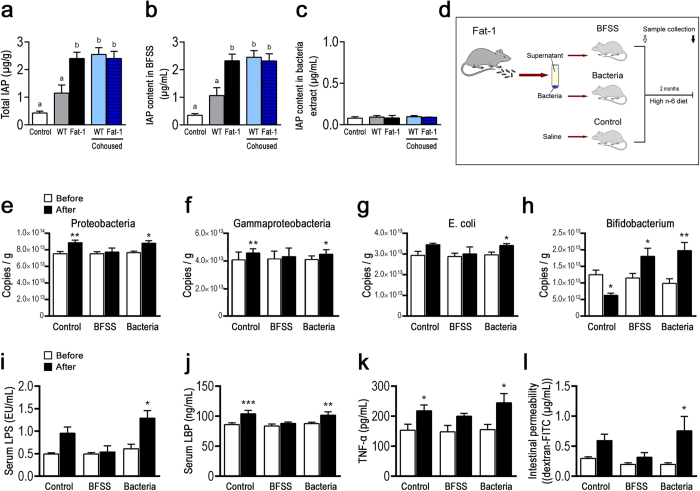
Fecal IAP from fat-1 mice can transmit to WT mice and alter their gut microbiota, metabolic endotoxemia and inflammation. Fresh feces collected from the separately housed WT and fat-1 (n = 10/group) and co-housed WT and fat-1 mice (n = 4/group, 2 WT+2 fat-1 in a cage) were separated into bacteria-free stool supernatant (BFSS) and bacterial pellet fractions. The levels of IAP in the whole stool **(a)**, BFSS **(b)** and bacterial pellets **(c)** were measured using an IAP assay kit. **(d)** Schema showing the animal groups and treatments. The BFSS and bacterial pellets from fat-1 mice were transferred by daily gavage or drinking water into WT mice (n = 5) that were simultaneously given a high n-6 diet. Two months after the treatments, the animals were subjected to analysis for changes in LPS production-related bacterial groups in the stool **(e–h)**, markers of endotoxemia **(i, j)** and inflammation **(k)**, and intestinal permeability **(l)**. Data are expressed as mean ± SE. Data with different superscript letters **(a–c)** are significantly different (P < 0.05) according to one-way ANOVA with Tukey test. Before and after treatment values **(e–l)** were analyzed by using paired T-test. *P < 0.05; **P < 0.01.

Based on our observations that fat-1 mice present a LPS-suppressing gut microbiota profile and that the LPS-promoting gut microbiota profile of the WT mice can be improved through co-housing with fat-1 mice, we hypothesize that fat-1 mice produce transmittable factors from the intestinal tissue that can modify the gut microbiota profile, leading to suppression of LPS production. We examined the levels of several endogenous antimicrobial peptides involved in regulating the gut microbiota (Supplementary Fig. S9), particularly the intestinal alkaline phosphatase (IAP), which is known to maintain normal gut microbial homeostasis^25^, suppress *E. coli* growth^26^,^27^,^28^,^29^,^30^, and detoxify LPS and luminal extra cellular ATP^26^,^31^. Our results showed that IAP expression in both mRNA and protein levels, histological staining of IAP, total IAP activity, and LPS-dephosphorylating activity were all significantly greater in the small intestine of fat-1 mice, compared to WT mice (Fig. 4). It is notable that the IAP isozyme AKP6 is up-regulated in WT mice, which can occur when the primary IAP isozyme AKP3 is down-regulated^32^. Interestingly, after long-term co-housing with fat-1 mice, WT mice also exhibit similarly high levels of IAP (Fig. 5a–c). These results suggest that it is IAP that is produced in the fat-1 mice and transmitted to the WT mice through the stool.

If IAP is a transmissible factor produced by the host tissue, then it is expected that transfer of bacteria-free stool supernatant (BFSS) from fat-1 to WT mice would generate a fat-1-like gut microbiota profile in the WT mice. Indeed, following two months of treatment with the IAP-containing BFSS from the fat-1 mice (Fig. 5d), we found that the BFSS-treated WT mice were able to prevent the previously observed increases of LPS-producing *Proteobacteria*, including *gammaproteobacteria*, *Enterobacteriaceae*, and *E. coli* (Fig. 5e-g and Supplementary Fig. S10) on a high n-6 PUFA diet, while increasing the abundance of LPS-suppressing *Bifidobacterium* (Fig. 5h) and decreasing markers of endotoxemia (Fig. 5i,j) and inflammation (Fig. 5k), as well as intestinal permeability (Fig. 5l). In contrast, administration of the bacterial pellet from the fat-1 mice failed to produce the same effects in the WT mice (Fig. 5e-l). These results indicate that the changes in WT mice following fecal transfer were induced by IAP in the stool of fat-1 mice, rather than the stool bacteria.

**Figure 6:**
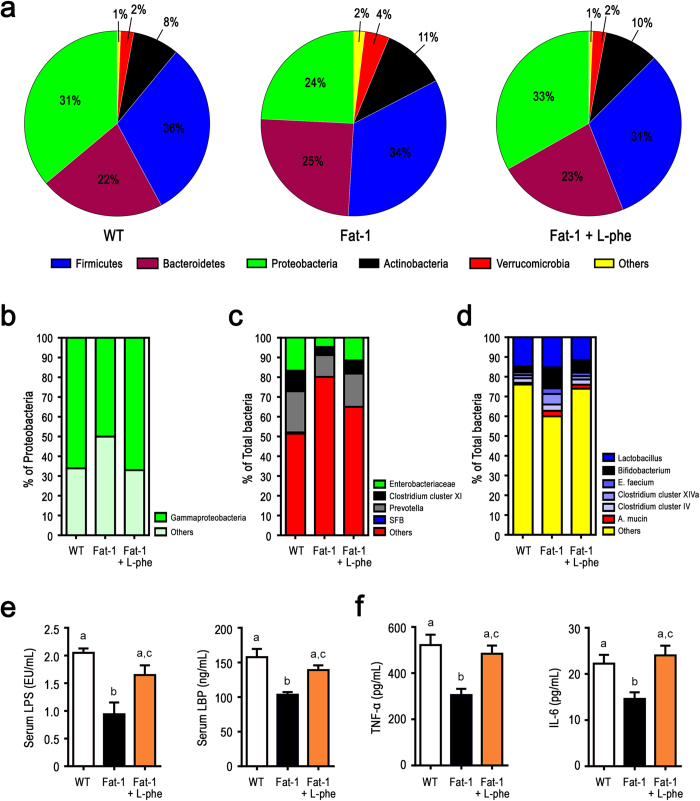
Inhibition of IAP eliminates the beneficial effects of elevated tissue n-3 PUFA on gut microbiota, metabolic endotoxemia, and inflammation. An IAP-specific inhibitor (10 mM L-phenylalanine) was added to the drinking water of a subgroup of fat-1 mice for two months. The treated fat-1 mice (n = 5) together with untreated fat-1 (n = 5) and WT mice (n = 5) were subjected to analysis for differences in gut microbiota and metabolic markers. (**a**) Relative abundance of the 5 most dominant phyla in the stool between groups. (**b**) Relative abundance of major classes of LPS-producing members of the *Proteobacteria* phylum between groups. (**c**) Relative abundance of major groups of LPS-producing and/or pro-inflammatory bacteria at the sub-phylum level. (**d**) Relative abundance of major groups of LPS-suppressing and/or anti-inflammatory bacteria at the sub-phylum level. (**e**) Metabolic parameters, including serum LPS and LBP levels. (**f**) Markers of chronic low-grade inflammation, including TNF-α and IL-6. Specific primers used are shown in Supplementary Table 3. For panels **e** & **f**, data are expressed as mean ± SE and data with different superscript letters are significantly different (P < 0.05) according to one-way ANOVA with Tukey test.

To further verify whether IAP is the primary factor responsible for the differences in the gut microbiota between fat-1 and WT mice, we examined the effects of IAP inhibition using L-phenylalanine, a specific non-competitive inhibitor^26^,^33^, on the status of gut microbiota, metabolic endotoxemia, and inflammation in fat-1 mice. After 8 weeks of treatment, we found that the L-phenylalanine-treated fat-1 group exhibited a significant increase in LPS-producing bacteria and a decrease in LPS-suppressing bacteria (Fig. 6a-d and Supplementary Fig. S11) compared to the untreated fat-1 group. Accordingly, the L-phenylalanine-treated fat-1 group showed significantly higher levels of markers of metabolic endotoxemia (Fig. 6e) and inflammation (Fig. 6f) compared to the untreated fat-1 group. These results support the notion that reducing the tissue n-6/n-3 PUFA ratio is able to alter the gut microbiota through increasing endogenous IAP activity.

### Omega-3 PUFA supplementation reduces metabolic endotoxemia

**Figure 7:**
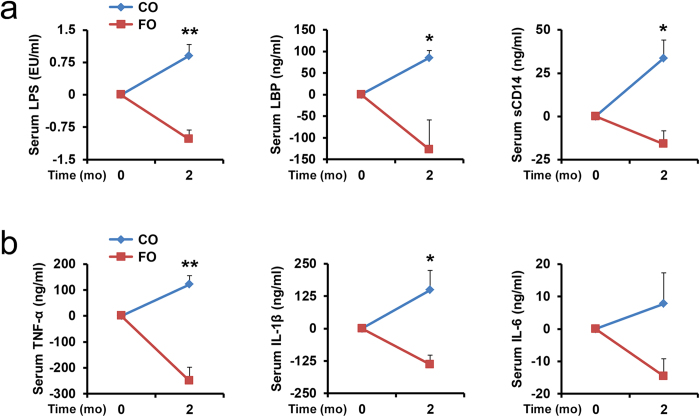
Fish oil supplementation suppresses metabolic endotoxemia and low grade inflammation. Twenty-month-old WT mice previously received a high n-6 PUFA (10% corn oil) diet were supplemented with n-3 PUFA (5% corn oil+5% fish oil) for 2 months. Serum samples were collected before and after the supplementation from the supplemented and control mice and subjected to analysis for markers of metabolic endotoxemia (LPS, LBP, sCD14) **(a**) and chronic inflammation (TNF-α, IL-1β, IL-6) (**b**). Data are expressed as mean ± SE. Significance was determined by unpaired two-tailed student T-test. *P < 0.05; **P < 0.01; ***P < 0.001. CO, corn oil; FO, fish oil.

Finally, we tested whether supplementing WT mice with n-3 PUFA can produce effects similar to those observed in the fat-1 mice. Aged (20-month-old) WT mice maintained on a high n-6 PUFA corn oil (CO) diet since weaning were switched to an n-3 PUFA-enriched fish oil (FO) diet for 2 months. After n-3 PUFA supplementation, the mice exhibited tissue essential fatty acid profiles comparable to those of the fat-1 transgenic mice (Supplementary Fig. S12). We found that the WT mice supplemented with FO showed decreased levels of LPS-producing and/or pro-inflammatory bacteria and increased levels of LPS-suppressing and/or anti-inflammatory bacteria (Supplementary Fig. S13), as well as significantly decreased markers of metabolic endotoxemia (Fig. 7a) and inflammation (Fig. 7b and Supplementary Fig. S14), while the WT mice on a CO diet showed increased levels of the markers. These results indicate that n-3 PUFA supplementation is effective in modifying the gut microbiota and reducing metabolic endotoxemia and inflammation.

## Discussion

**Figure 8:**
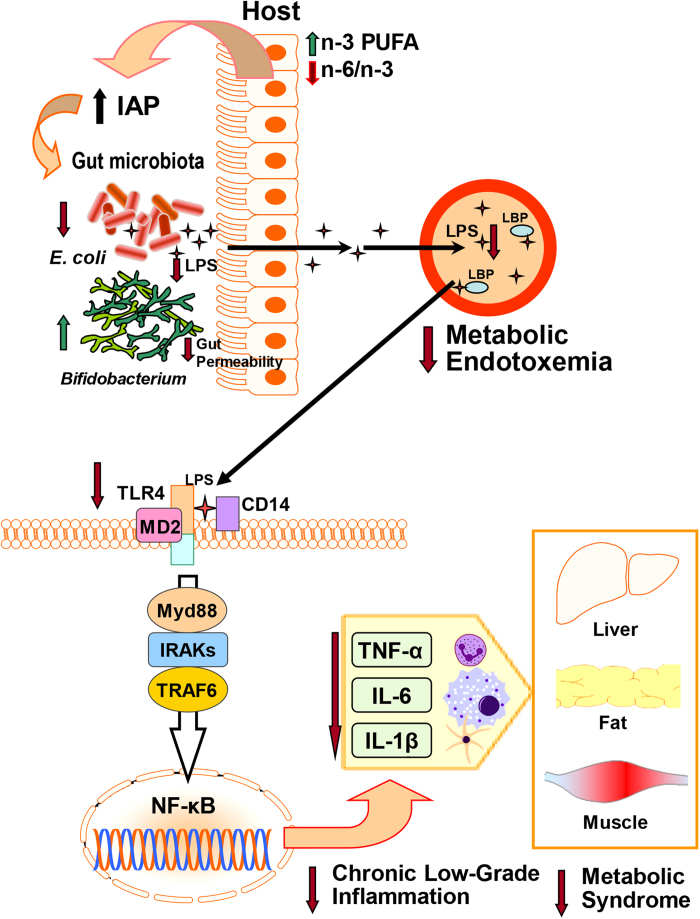
Diagram illustrating a proposed mechanism by which elevated tissue n-3 PUFA status and a reduced tissue n-6/n-3 fatty acid ratio up-regulate endogenous IAP activity in the gut, decreasing LPS-producing bacteria (e.g. E. coli) while increasing LPS-suppressing bacteria (e.g. Bifidobacterium). These changes lower LPS production and gut permeability, resulting in reduced metabolic endotoxemia. The subsequent reduction of inflammatory cytokines leads to the suppression of chronic low-grade inflammation and metabolic syndrome.

Metabolic endotoxemia derived from gut dysbiosis is central to the pathogenesis of chronic low-grade inflammation, an underlying factor of aging as well as many modern chronic diseases^4^,^6^. Understanding the factors that contribute to metabolic endotoxemia and identifying safe and effective means of limiting its development is critical for the prevention and treatment of these diseases. The worldwide trends of excessive n-6 PUFA and insufficient n-3 PUFA intake have been implicated in today’s health epidemics^17^. However, the connections between the shifts in dietary essential fatty acid content and the incidence of chronic disease remain to be elucidated. The present study demonstrates for the first time, using a transgenic mouse model, that n-6 and n-3 PUFA exert opposing effects on metabolic endotoxemia by modulating the endogenous expression of IAP and thereby modifying the gut microbiota, leading to differential outcomes in chronic low-grade inflammation and metabolic syndrome (Fig. 8).

Although recent dietary supplementation studies have shown some effects of n-6 and n-3 PUFA on gut microbiota^34^,^35^,^36^,^37^,^38^, they are unable to distinguish whether the effects arise from interactions between the gut microbiota and the dietary components or from changes in the host tissue fatty acid composition. In this study, we altered the host tissue fatty acid composition through a genetic modification. Our genetic approach using the *fat-1* transgenic mice allowed us to eliminate potential confounding factors of diet, so that any differences between the transgenic and WT mice could be attributed to the changes in the host tissue essential fatty acid composition. As reported in the present study, the significant differences in gut microbiota profiles between fat-1 and WT mice, despite being maintained on the same diet, can be attributed to their respective tissue essential fatty acid composition. This study is thus the first to provide evidence that the alteration of host tissue fatty acid composition, rather than direct interaction of dietary fats with gut bacteria, is primarily responsible for the effects of essential fatty acids on the gut microbiota.

IAP is an endogenous antimicrobial peptide with numerous physiological functions^39^,^40^. It is highly expressed in the small intestine, secreted from apical enterocytes into the lumen in microvilli vesicles, and travels to the large intestine^27^. IAP is known to inhibit the growth of *E. coli* and Gram-negative bacteria by dephosphorylating LPS located in the outer membrane^26^,^27^,^28^,^29^,^30^. IAP is also able to dephosphorylate ATP^31^, which has been shown to reduce the survival of gram-positive bacteria^31^ and support the growth of gram-negative bacteria such as *E. coli*^32^. Oral IAP supplementation has also been shown to prevent *E. coli* overgrowth^41^. IAP is therefore a major enzyme of interest for its gut microbiota-modifying properties. As IAP is resistant to intestinal degradation by host digestive enzymes, the measurement of IAP and its counterpart, LPS-dephosphorylating capacity, in the feces of mice has been shown to reliably reflect its endogenous activity^42^. It is clear that IAP expression in the intestine is a critical determinant of the gut microbiota profile. With evidence from gene expression to enzyme activity, our study has thoroughly demonstrated that IAP is up-regulated in a host tissue environment with increased n-3 PUFA content, as seen with transgenic conversion of n-6 to n-3 PUFA or dietary n-3 PUFA supplementation. Together with our data from co-housing, fecal transfer, and IAP inhibition experiments, our findings indicate that IAP is a primary factor mediating the effects of tissue essential fatty acid composition on gut microbiota.

The mechanisms by which increased tissue n-3 PUFA content may modulate IAP expression remain to be further investigated. Previous studies have shown that fish oil supplementation alters the fatty acid composition of the intestinal brush border membrane and increases IAP activity, possibly by modulating membrane fluidity^43^ or the local lipid microenvironment^44^. A recent study has also reported that IAP expression can be induced by resolvin E1^26^, which is a lipid mediator derived from the n-3 PUFA eicosapentaenoic acid (EPA) and is known to be elevated in our fat-1 transgenic mice^45^. It is possible that multiple mechanisms are involved in the regulation of IAP expression by n-3 PUFA.

Metabolic endotoxemia can be determined by the abundance of bacteria affecting LPS production and gut barrier function. It is therefore conceivable that the marked reductions in LPS-producing and/or pro-inflammatory bacteria (e.g. *Proteobacteria*) and increases in LPS-suppressing and/or anti-inflammatory bacteria (e.g. *Bifidobacterium*) observed in the fat-1 mice result in significant suppression of endotoxemia and inflammation. Recent studies have shown that LPS-producing bacteria are abundant in obese subjects with type 2 diabetes^8^,^46^,^47^. It has also been shown that mice fed a 5% corn oil diet for one week exhibit overgrowth of *Proteobacteria*^48^. Along these lines, the 10% corn oil diet used in the present study was also able to induce dramatic increases in LPS-producing bacteria, including *Proteobacteria*, in WT mice (Fig. 3). In this context, decreasing the abundance of LPS-producing bacteria is a key mechanism for the reduction of metabolic endotoxemia. Another potential mechanism contributing to the reduction of serum LPS may be a decrease in gut permeability, due to the observed elevation of gut barrier-protecting bacteria such as *Bifidobacterium* and the up-regulation of tight junction proteins and its adaptor molecules by a reduced n-6/n-3 PUFA ratio (Supplementary Fig. S6).

It is interesting to note that certain factors related to inflammation and metabolic syndrome, namely IL-1β and serum triglycerides, remained significantly different between WT and fat-1 mice following the antibiotic treatment and co-housing experiments (Fig. 2 and Supplementary Figs. S3d,S4d). These results suggest that IL-1β and plasma TG, unlike many of the other measured factors, are not dependent on the gut microbiota profile or more specifically, the reduction of LPS-associated endotoxemia. Instead, they are likely modulated primarily by other pathways. In fact, IL-1β is known to be down-regulated by n-3 PUFA through suppression of NLRP3 inflammasome activity^49^, while plasma TG can be lowered by n-3 PUFA through reduction of hepatic lipogenesis and other mechanisms^50^. Thus, the present study reveals that many of the beneficial effects of elevated tissue n-3 PUFA status on inflammation and metabolic syndrome are dependent on the modulation of the gut microbiota, but that the lowering effects of n-3 PUFA on IL-1β and plasma TG appear to be gut microbiota-independent. This gut microbiota-related phenomenon provides insight into the diverse responses of individuals to n-3 PUFA supplementation, given the individual variation among gut microbiota profiles.

From a translational standpoint, we utilized n-3 PUFA supplementation in this study to confirm that the elevated tissue n-3 PUFA status and the beneficial effects observed in the fat-1 mice could also be achieved through dietary intervention. Our discovery that elevating tissue n-3 PUFA status and lowering the n-6/n-3 PUFA ratio can improve the gut microbiota profile and suppress chronic low-grade inflammation provides two major implications for today’s health problems. On one hand, this study highlights the excess of n-6 PUFA and deficiency of n-3 PUFA in the Western diet^17^,^18^ as a cause of modern health epidemics. On the other hand, this study points to a new strategy for the prevention and treatment of chronic diseases by reducing the tissue n-6/n-3 PUFA ratio through n-3 PUFA supplementation and reducing n-6 PUFA intake. Furthermore, the mediating role of IAP in the pathway explored in this study supports the use of exogenous IAP as a potential therapy for inflammatory diseases^40^. Finally, given that gut dysbiosis and metabolic endotoxemia are often linked to many clinical problems, the capability of n-3 PUFA to reverse these conditions indicates the potential of n-3 PUFA supplementation as a therapeutic means for treating such cases, and its efficacy could be evaluated through the measurement of LPS and related factors as biomarkers.

## Methods

### Animals and diets

Transgenic *fat-1* mice were generated as described previously^22^ and backcrossed onto a C57BL/6 background. Heterozygous *fat-1* mice were then mated to obtain wild-type (WT) and *fat-1* littermates. The phenotype of the *fat-1* mice (as indicated by increased tissue n-3 PUFA) was confirmed by fatty acid analysis using gas chromatography (GC) and the mice were bred at the Massachusetts General Hospital (MGH) animal facility. In addition, a subset of 8-month-old male C57BL/6 WT mice was purchased from Charles River Laboratories. Mice were housed in a biosafety level 2 room in hard top cages with two or three mice per cage. Mice were maintained in a temperature-controlled room (22–24 °C) with a 12-h light/12-h dark diurnal cycle, and allowed for food and water ad libitum. Normally, mice were maintained on a chow diet (Laboratory Rodent Diet 5001) from LabDiet. Fat-1 mice and their WT littermates were fed a diet high in n-6 PUFA (AIN-76A with 10% corn oil) from LabDiet. The group of mice supplemented with n-3 PUFA was fed an AIN-76A diet with 5% corn oil and 5% fish oil, from Harlan Laboratories. Supplementary Table S2 shows the detailed nutrient composition and n-6/n-3 fatty acid profiles of all diets used. All animal procedures in this study were carried out in accordance with the guidelines approved by the MGH Subcommittee on Research Animal Care.

### Animal experiments

Body weight and food intake were measured every week or other week for all mice. For collection of blood samples, mice were fasted for 6 h during the light phase period and blood was taken from the facial vein unless otherwise specified. Animals were euthanized by i.p. injection of pentobarbital (200 mg/kg). Several experiments with special treatments are described below.

### Determination of metabolic endotoxemia, chronic low grade inflammation and gut microbiota in fat-1 and WT mice

Male WT (n = 10) and fat-1 (n = 10) littermates were maintained on a high n-6 PUFA diet (AIN-76A with 10% corn oil) for 8 months after weaning. Another group of male 8-month-old WT mice (n = 10) fed a chow diet since weaning were used as control. Also, a subset of WT (n = 7) and fat-1 (n = 5) littermates were maintained on a high n-6 PUFA diet (AIN-76A with 10% corn oil) for 20 months after weaning. Both 8-month and 20-month-old mice were subjected to analysis of markers of metabolic endotoxemia (including LPS, LBP, and sCD14), systemic chronic low grade inflammation (including TNF-α, IL-1β, IL-6, MCP-1 and IL-10) and metabolic syndrome (including weight gain, fasting blood glucose and insulin, insulin resistance index assessed by HOMA-IR) and analysis of fecal microbiota by qPCR and stool culture. The mice were then euthanized after 6 hrs fasting and the intestines were cut open longitudinally and flushed twice with PBS to remove luminal content. Portions of the small and large intestines, liver, and intestinal luminal contents were stored at −80 °C for further analysis.

### Antibiotic (ABX) experiments

Ten-month-old male WT (n = 10) and fat-1 (n = 10), maintained on the same high n-6 diet after weaning, were divided into two groups for each genotypes (n = 5/group). One group from each genotype received a broad spectrum antibiotic cocktail (ABX) containing ampicillin (1 g/L), vancomycin (500 mg/L), neomycin sulfate (1 g/L) (added to drinking water) and metronidazole (100 mg/kg) (orally gavaged every 12 h) for 6 weeks to make a macroscopically germ free mice^51^. Validation of successful depletion of gut microbiota after the antibiotic treatment was performed as described previously^51^. Mice with or without antibiotics treatment were subjected to analysis for markers of metabolic endotoxemia and systemic chronic low grade inflammation and serum lipid profile (including total cholesterol, triglyceride, LDL and HDL).

### Co-housing experiments

Male fat-1 (n = 4) and WT littermates (n = 4) were co-housed in two cages (2 mice from each genotype per cage) and fed an identical high n-6 diet for 8 months after weaning. For comparison, another 10 WT and 10 fat-1 male mice were housed separately and fed the same diet for the same period of time. In addition, one other group of male WT mice (n = 10) fed a chow diet was used as a control for stool IAP analysis. During the co-housing, the mice changed their partners and switched cages several times to rule out a stochastic cage effect^52^. After the 8-month co-housing or separate housing, all groups of animals were subjected to analysis for markers of metabolic endotoxemia, chronic low-grade inflammation, and metabolic syndrome, as well as analysis of fecal microbiota and IAP.

### Fecal transfer experiments

Fresh fecal matter was collected from male 9-month-old fat-1 mice and subjected to a serious of centrifugation steps to separate the feces into bacteria-free stool supernatant (BFSS) and bacterial pellets as described previously^53^. BFSS was then subjected to heat by treating at 95°C for 30 min to kill residual bacteria^54^. After heating, bacterium-free status was confirmed by culturing BFSS for aerobic and anaerobic bacteria. The presence of IAP in BFSS was confirmed by IAP assay (See *Bacterial culture* and *IAP assay* sections below). A portion of bacterial pellets was further processed to make bacterial extracts^53^ for measurement of IAP. Male WT mice previously maintained on a chow diet for 10 months after weaning were separated into three groups (n = 5 for each group) and gavaged with either 500 μl of normal saline (control) or BFSS twice a day. As the IAP levels in the BFSS obtained from the fat-1 mice were in the range of 1900-2450 ng/ml, WT mice were gavaged with ~2000 ng of IAP daily. The third group was given bacterial pellets mixed in the drinking water. Fresh bacterial pellets (~200 mg) were suspended in 250 ml of sterile water, containing approximately 6 ×10^5^/ml bacteria^55^. The treated water was changed twice a week. During the two-month treatment, the three groups of animals were maintained on a high n-6 diet (AIN-76A with 10% corn oil). After treatment for 2 months, the mice were subjected to analysis for markers of metabolic endotoxemia and chronic low-grade inflammation, as well as analysis of fecal microbiota and IAP.

### Determination of the effect of inhibiting endogenous IAP activity in the fat-1 mice

Male 10-month-old WT mice (n = 5) and fat-1 mice (n = 10), all maintained on a high n-6 diet since weaning, were used for this experiment. The fat-1 mice were divided into two groups (n = 5 per group). One of the fat-1 groups and the WT group received normal drinking water, while the other fat-1 group was given drinking water containing 10 mM L-Phenylalanine (L-Phe) (Sigma, USA), a well-studied specific inhibitor of IAP^26^, for 2 months. The three groups of mice were then subjected to serum and stool analysis as described above.

### Bacterial culture for fecal microbiota analysis

Bacterial culture was performed as described previously^33^,^56^. Individual stool samples were collected directly in microfuge tubes containing 200 μl of Brain Heart Infusion (BHI) media and kept on ice. The weight of each stool sample was determined. Additional BHI media was added to each tube obtaining a specific weight/volume ratio (100 μl BHI per 10 mg stool sample). Samples were vortexed and serially diluted, then plated on MacConkey agar (BD, USA) or Bifido agar (Anaerobe Systems, Morgan Hill, CA) to enumerate *Enterobacteriaceae* and *Bifidobacterium*, respectively. BFSS was cultured on BHI agar (BD, USA) and Brucella agar plates (Anaerobe Systems, Morgan Hill, CA) and BMacConkey agar plates for aerobic and anaerobic bacteria, respectively. BHI and MacConkey agar plates were incubated in ambient air for 24 h at 37 °C, and Brucella and Bifido agar plates were incubated in an anaerobic bag (Fisher Scientific, Hampton, NH) in a 37 °C incubator for 48 hrs. Duplicate plate values were averaged and bacterial densities were expressed as the number of colony forming units (CFU)/g wet weight of feces.

### Quantitative analysis of gut microbiota

*a) Extraction and purification of DNA from fecal samples*. Bacterial genomic DNA was extracted from fresh stool samples (~100–180 mg) using the QIAamp DNA Stool Mini Kit (Qiagen, Valencia, CA), following the manufacturer’s instructions. In order to increase its effectiveness, the lysis temperature was increased to 95 °C. The eluted DNA was treated with RNase, concentration was determined by absorbance at 260 nm (A260), and purity was estimated by determining the A260/A280 ratio with a Nanodrop spectrophotometer (Biotek, Winooski, VT), diluting to 20 ng/μl and then storing at −20 °C until analysis. *b) Bacterial DNA measurement by 16S rRNA gene-based real-time Quantitative PCR (qPCR).* Microbial profiling of feces was performed as previously described^57^. Briefly, qPCR was performed with a PRISM 9000 Light Cycler (Applied Biosystems, USA) using the iTaq universal SYBR Green Supermix (Bio-Rad, USA) and group-specific primers listed in Supplementary Table 3. The specificity of the primers and the limit of detection were determined as previously described^58^. Samples and the standards were run in duplicates with a total volume of 20 μl/well containing 500 nM primer and 40 ng template gDNA. Amplification programme and data acquisition waere performed following the protocol provided with SYBR Green (Bio-Rad, USA). A genomic DNA standard from reference strains (BEI Resources, Manassas, VA) as in Supplementary Table S3, was converted into 16S rRNA copy numbers and serially diluted to generate a standard curve. The 16S rRNA copy number was determined for each genomic standard by first converting the genomic standard quantities into genome copy numbers and then into 16S rRNA copy numbers, as described previously^57^. Threshold cycle values from qPCR for the test samples were used to determine their corresponding numbers of 16S rRNA gene copies based on the standard curve. Data is expressed as 16S rRNA gene copy number per gram of stool as well as relative abundance of percentage of total bacteria at sub-phylum level bacterial groups.

### Gene expression analysis by semi-quantitative PCR

Total RNA was isolated from tissue samples of small intestine and liver tissues (~50 mg) using TRIzol reagent (Invitrogen Life Technologies, Grand Island, NY), following the manufacturer’s instructions. RNA concentrations and purity were estimated by determining the A260/A280 ratio with a Nanodrop spectrophotometer (Biotek). Reverse transcription of mRNA was performed using the iScript cDNA Synthesis Kit (Bio-Rad). qPCR was carried out using SYBR Green in a PRISM 9000 Light Cycler (Applied Biosystems), following the manufacturer’s instructions. All primers (Supplementary Table S4) were synthesized by Invitrogen. All samples were processed in triplicate and normalized to β-actin. The expression levels were calculated using the ΔΔCt method after correcting for differences in PCR efficiencies, and values were expressed relative to those of the control WT group.

### Measurement of LPS concentration

Serum LPS concentrations were measured with a ToxinSensor Chromogenic Limulus Amebocyte Lysate (LAL) Endotoxin Assay Kit (GenScript), following the manufacturer’s instructions. Briefly, samples were diluted 10- to 50-fold with endotoxin-free water, adjusted to the recommended pH, and heated for 10 min at 70 °C to minimize inhibition or enhancement by contaminating proteins. LAL reagents were added to serum and incubated at 37 °C for 45 min, and the absorbance was read at 545 nm. A spiked control at 0.45 EU/ml was performed for each sample to check that no significant inhibition or activation occurred.

### Measurement of intestinal alkaline phosphatase (IAP) level and activity

Small intestinal IAP specific activity (as it relates to protein) was measured as previously described^33^ and expressed as picomoles pNPP hydrolyzed/min/μg of protein. IAP levels in the BFSS or bacterial extracts were measured by using a SensoLyte pNPP Alkaline Phosphatase Assay Kit (AnaSpec, CA, USA) according to manufacturer’s instructions. Briefly, 50 ul of bacterial free stool supernatant, bacterial extracts, and IAP standards were mixed with 50 μl of phosphatase assay reagent (p-nitrophenyl phosphate (pNPP)), incubated for 1 hr, and the optical density was determined at 405 nm. IAP levels were expressed as μg/g sample.

### Western blotting analysis of IAP

Western blotting was performed as previously described^59^. Briefly, proximal small intestine tissues were harvested and cut open longitudinally, and luminal contents were removed. The tissues were washed with PBS and homogenized with liquid nitrogen, and homogenates were mixed with radioimmunoprecipitation assay (RIPA) buffer (50 mM Tris-HCl, pH 7.4, 150 mM NaCl, 1% Triton X-100, 1% sodium deoxycholate, 0.1% sodium dodecyl sulfate) containing protease inhibitor cocktail (Sigma Aldrich), incubated on ice for 30 min, and centrifuged at 14,000 g for 10 min at 4 °C. The supernatant was collected and protein concentration was quantified by the Coomassie blue protein assay (Thermo Scientific, Rockford, IL, USA) using bovine serum albumin (BSA) as the standard. Proteins (30 μg) were resolved on SDS-PAGE gels and transferred onto nitrocellulose membranes (Osmonics, Minnetonka, MN, USA). The membranes were blocked with 5% nonfat dry milk in Tris-buffered saline with 0.05% Tween 20 (TBS-T) for 1 h at room temperature and then probed with IAP primary antibodies (GTX27322, GeneTex, San Antonio, TX, USA) in 5% nonfat dry milk in TBS-T at 4 °C overnight. After washing three times in TBS-T, the blots were further incubated with the corresponding secondary antibodies conjugated with horse radish-peroxidase for 1 h at room temperature (Santa Cruz Biotechnology, Santa Cruz, CA). Chemiluminescence was detected with Pierce ECL Western blotting substrate (Thermo Scientific, Rockford, IL, USA) and visualized by ChemiDoc MP Imaging System (Bio-Rad, Hercules, CA, USA).

### Immunofluorescent staining of IAP

Small intestinal tissue sections on slides were permeabilized and fixed in 100% methanol at 4 °C for 30 minutes. The tissues were washed three times and incubated with the blocking solution (5% BSA in PBS). The tissues were then incubated with the IAP primary antibodies (GTX27322, GeneTex) for 2 hrs or overnight, washed three times with PBS containing 0.1% Tween-20 for 15 minutes, and finally incubated with secondary antibodies conjugated to Alexa-598 (A21207, Invitrogen) and Hoechst stain to detect nuclear DNA (H1399, Invitrogen) for 30 minutes. The slides were washed extensively with PBS and mounted with Immu-mount (Fisher Scientific). All matched samples were photographed using an immunofluorescence microscope (LSM710, Zeiss). All pictures were taken with the same exposure conditions without autoscaling.

### Measurement of cytokine levels and other circulating factors

Serum samples were analyzed for levels of TNF-α, IL-1β, IL-6, MCP-1, and IL-10 by Bio-Plex immunoassays formatted on magnetic beads (Bio-Rad), following the manufacturer’s instructions. Xponent software (Luminex, Austin, TX) was used for data acquisition and analysis. ELISA kits were used to analyze serum levels of sCD14 (MyBioSource, San Diego, CA), insulin (Crystal Chem, Downers Grove, IL), and LBP (NeoBioLab, Cambridge, MA), according to the manufacturers’ instructions.

### Determination of fatty acid composition of mouse tissues and diets

Fatty acid profiles of mouse diets, tail and intestinal tissues were analyzed by gas chromatography as described previously^60^. Briefly, tissue or food samples were grounded to powder under liquid nitrogen and subjected to total lipid extraction and fatty acid methylation by 14% boron trifluoride (BF3)-methanol reagent (Sigma-Aldrich) at 100 °C for 1 h. Fatty acid methyl esters were analyzed using a fully automated HP5890 gas chromatography system equipped with a flame-ionization detector (Agilent Technologies, Palo Alto, CA). The fatty acid peaks were identified by comparing their relative retention times with the commercial mixed standards (NuChek Prep, Elysian, MN), and area percentage for all resolved peaks was analyzed by using a PerkinElmer M1 integrator.

### Measurement of intestinal permeability

An intestinal permeability assay was performed as previously described^33^. Briefly, mice were gavaged with a phosphate buffer saline (PBS, pH 7.2) containing FITC-dextran (70 kDa, Sigma-Aldrich) at a dose of 600 mg/kg body weight. Blood samples (120 μL) were collected after 90 minutes from the facial vein. Serum was diluted with an equal volume of PBS, and fluorescence intensity was measured using a fluorospectrophotometer (Perkin-Elmer) with an excitation wavelength of 480 nm and an emission wavelength of 520 nm. Serum FITC-dextran concentration was calculated from a standard curve generated by serial dilution of FITC-dextran in PBS.

### Immunoglobulin A (IgA) measurement

Supernatant derived from mouse ileal content or stool was used to quantify total IgA by using a mouse IgA kit (Bethyl Laboratories, Inc, TX, USA) according to the manufacturer’s instructions.

### Measurement of tissue LPS dephosphorylating activity

Small intestinal LPS dephosphorylating activity was measured as described previously^34^. Briefly, small intestinal tissues were homogenized in 500 μl of homogenization buffer, centrifuged to remove insoluble material at 11,000 rpm for 3 min. To determine the protein concentrations a Bradford assay (Bio-Rad) was performed on the small intestinal samples, according to the manufacturer’s instructions. Forty μl of a 5 mg/ml solution of *Escherichia coli* 055:B5 LPS (Sigma, USA) were then added to 15 μl lysate and left for 2 h at room temperature. Free phosphate released in solution was measured by using the malachite green assay kit, according to the manufacturer’s instructions (Cayman Chemical Company, MI, USA).

### Plasma lipid profiles

Serum total cholesterol (TC) and triglycerides (TG) were measured by the Clinical Pathology Laboratory at the MGH Center for Comparative Medicine. Serum high-density lipoprotein cholesterol (HDL-C) was enzymatically determined using a kit from Pointe Scientific (Canton, MI) following the manufacturer’s instructions. The concentration of low-density lipoprotein cholesterol (LDL-C) was calculated using the Friedewald equation: LDL cholesterol = total cholesterol – HDL cholesterol–TG/5^33^, and the atherogenic index (TC-HDL-C/HDL-C) was calculated as described previously^61^.

### Statistical analysis

Data were expressed as mean ± SE. Statistical differences between more than two test groups were evaluated by one-way analysis of variance (ANOVA) with Tukey’s multiple comparison post-tests. The antibiotic and co-housing experiments were subjected to two-way ANOVA followed by Sidak’s multiple comparisons test. Data with two groups were analyzed by unpaired two-tailed student T-test and data gathered from before and after treatment were analyzed by repeated-measure, two-way ANOVA followed by Sidak’s multiple comparisons test. Data were checked for heterogeneous variance with the Bartlett’s and Brown-Forsythe tests or the F test. Significance was considered to be at *P *< 0.05. Statistical analyses were performed using GraphPad Prism version 6 (GraphPad Software, La Jolla, CA).

## Additional Information

**How to cite this article**: Kaliannan, K. *et al.* A host-microbiome interaction mediates the opposing effects of omega-6 and omega-3 fatty acids on metabolic endotoxemia. *Sci. Rep.* **5**, 11276; doi: 10.1038/srep11276 (2015).

## Supplementary Material

Supplementary Information
