# Supplementary material for: A host-microbiome interaction mediates the opposing effects of omega-6 and omega-3 fatty acids on metabolic endotoxemia
Source: Sci Rep. 2015 Jun 11;5:11276. doi: 10.1038/srep11276 (PMC4650612; doi:10.1038/srep11276)
Supplement: Supplementary Information [file srep11276-s1.pdf]

A host-microbiome interaction mediates the opposing effects of omega-6 and omega-3 fatty acids on metabolic endotoxemia

Kanakaraju Kaliannan, Bin Wang, Xiang-Yong Li, Kui-Jin Kim, Jing X. Kang*

Laboratory of Lipid Medicine and Technology, Department of Medicine, Massachusetts General Hospital and Harvard Medical School, Boston, MA 02129, USA

Supplementary Information

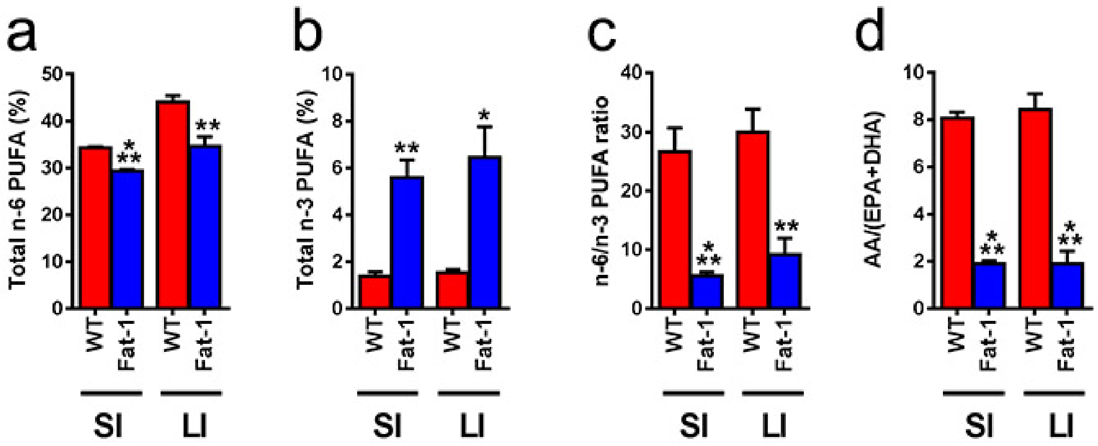

Supplementary Fig. S1. Differential fatty acid profiles of the small (SI) and large (LI) intestinal tissue from 8 month old WT (n=4), *Fat-1* transgenic mice (n=5). Percentages of total n-6 PUFA **(a)** and n-3 PUFA **(b)** and the ratios of tissue n-6/n-3 PUFA **(c)** and AA/(EPA+DHA) **(d)**, as determined by gas chromatography. Values are expressed as mean ± SE. Significance was determined by unpaired two-tailed student T-test. (*$P < 0.05$, **$P < 0.01$, ***$P < 0.001$).

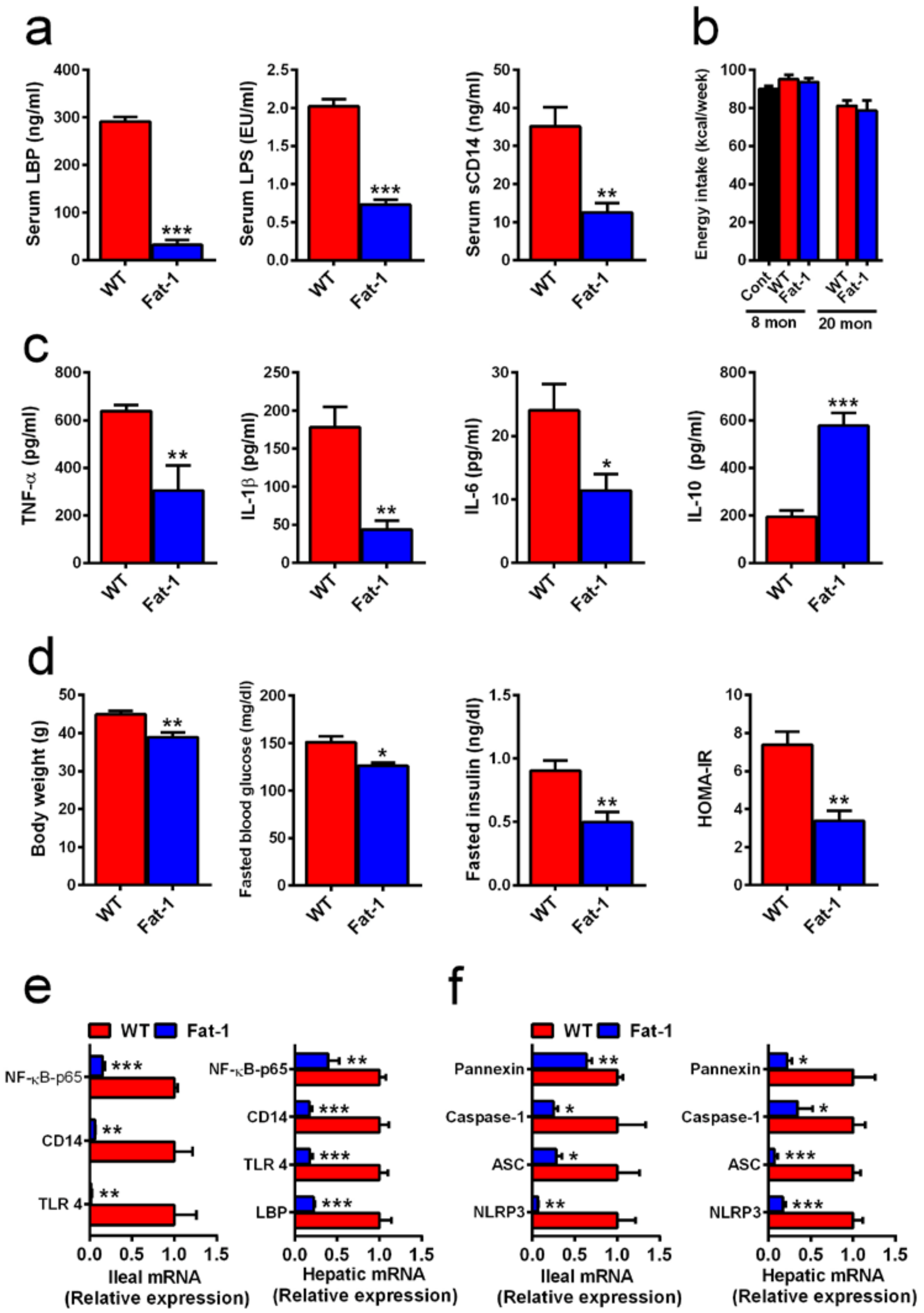

Supplementary Fig. S2. Endogenous conversion of omega-6 to omega-3 fatty acids reduces metabolic endotoxemia and systemic low grade inflammation in aged mice. Twenty month old WT (n=7) and fat-1 (n=5) littermates were maintained on a high n-6 (10% corn oil) after weaning. Blood and tissue samples were collected from all the mice at the same time and subjected to various analyses. **(a)** Parameters of metabolic endotoxemia **(**LPS, LBP and sCD14); **(b)** Energy intake; **(c)** Serum levels of inflammatory cytokines (TNF-α, IL-1β, IL-6 and IL-10) and **(d)** Markers of metabolic syndrome (body weight, fasting blood glucose, fasting insulin, and HOMA-IR); **(e)** Expression levels of LPS pathway related factors (LBP, CD14, Toll like Receptor 4 and NFκB-p65) in ileum and liver; **(f)** Expression levels of factors related to LPS-NLRP3 inflammasome pathway (NLRP3, ASC, Caspase 1 and Pannexin) in ileum and liver. Data are expressed as mean ± SE. Significance was determined by unpaired two-tailed student T-test. *$P < 0.05$; **$P < 0.01$; ***$P < 0.001$.

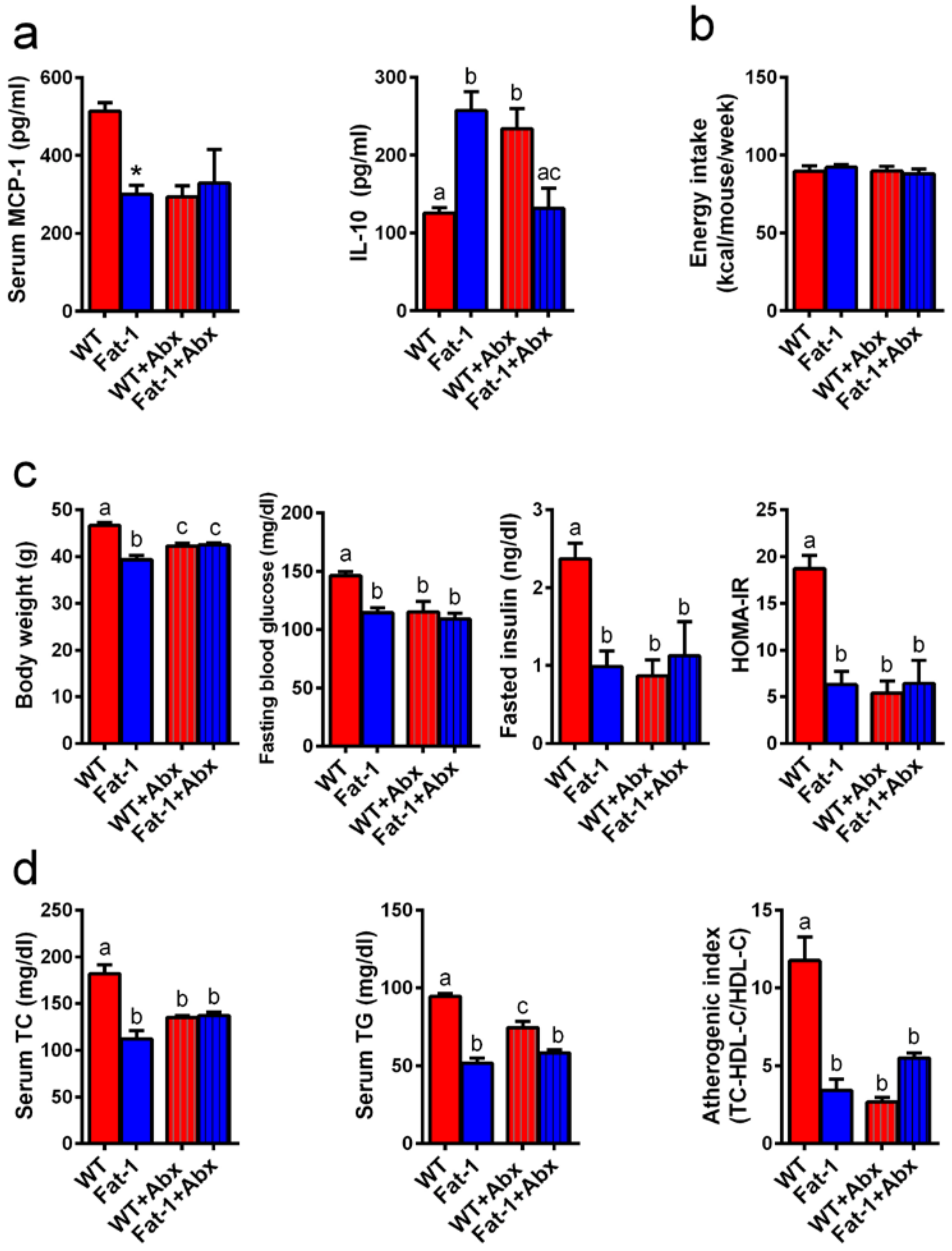

Supplementary Fig. S3. Antibiotic treatment alters the effects of tissue omega-6 and omega-3 PUFA status on metabolic endotoxemia, chronic low grade inflammation and metabolic syndrome. Separately housed male 10-month-old WT (n=10) and fat-1 (n=10) were maintained on the same high n-6 diet, and half of them in each group received a broad spectrum antibiotic cocktail (Abx) consisting of Ampicillin (1g/L), Vancomycin (500mg/L), Neomycin sulfate (1g/L) (added to drinking water) and Metronidazole (100mg/kg) (orally gavaged every 12 h) for about 2 months. **(a)** Serum MCP-1 and IL-10 levels; **(b)** Energy intake; **(c)** Metabolic parameters including body weight, fasting blood glucose, fasting serum insulin, and HOMA-IR; **(d)** serum lipid profile with atherogenic index. Data are expressed as mean ± SE. Data with different superscript letters are significantly different ($P < 0.05$) according to one-way ANOVA with Tukey Test.

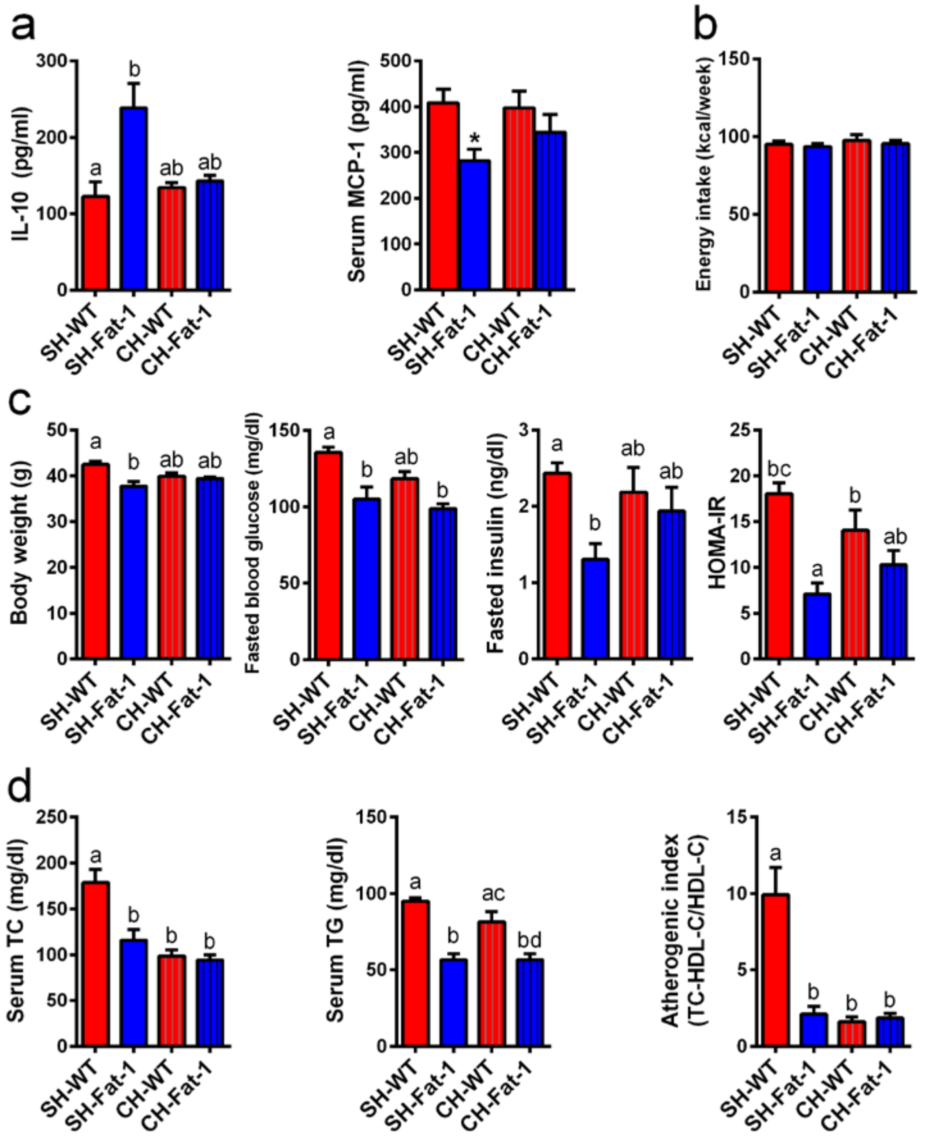

Supplementary Fig. S4. Co-housing of animals (WT+Fat-1) alleviates the differences between WT and fat-1 mice in metabolic endotoxemia, chronic low grade inflammation and metabolic syndrome. For the co-housing experiments, fat-1 (n=4) and WT littermates (n=4) were co-housed in two cages (2 mice from each genotype / cage) and fed an identical high n-6 diet for 8 months after weaning. **(a)** Serum MCP-1 and IL-10 levels; **(b)** Energy intake; **(c)** Metabolic parameters including body weight, fasting blood glucose, fasting serum insulin, and HOMA-IR, and **(d)** Serum lipid profile with atherogenic index. Data are expressed as mean ± SE. Data with different superscript letters are significantly different ($P < 0.05$) according to one-way ANOVA with Tukey Test. SH, separately housed; CH, co-housed.

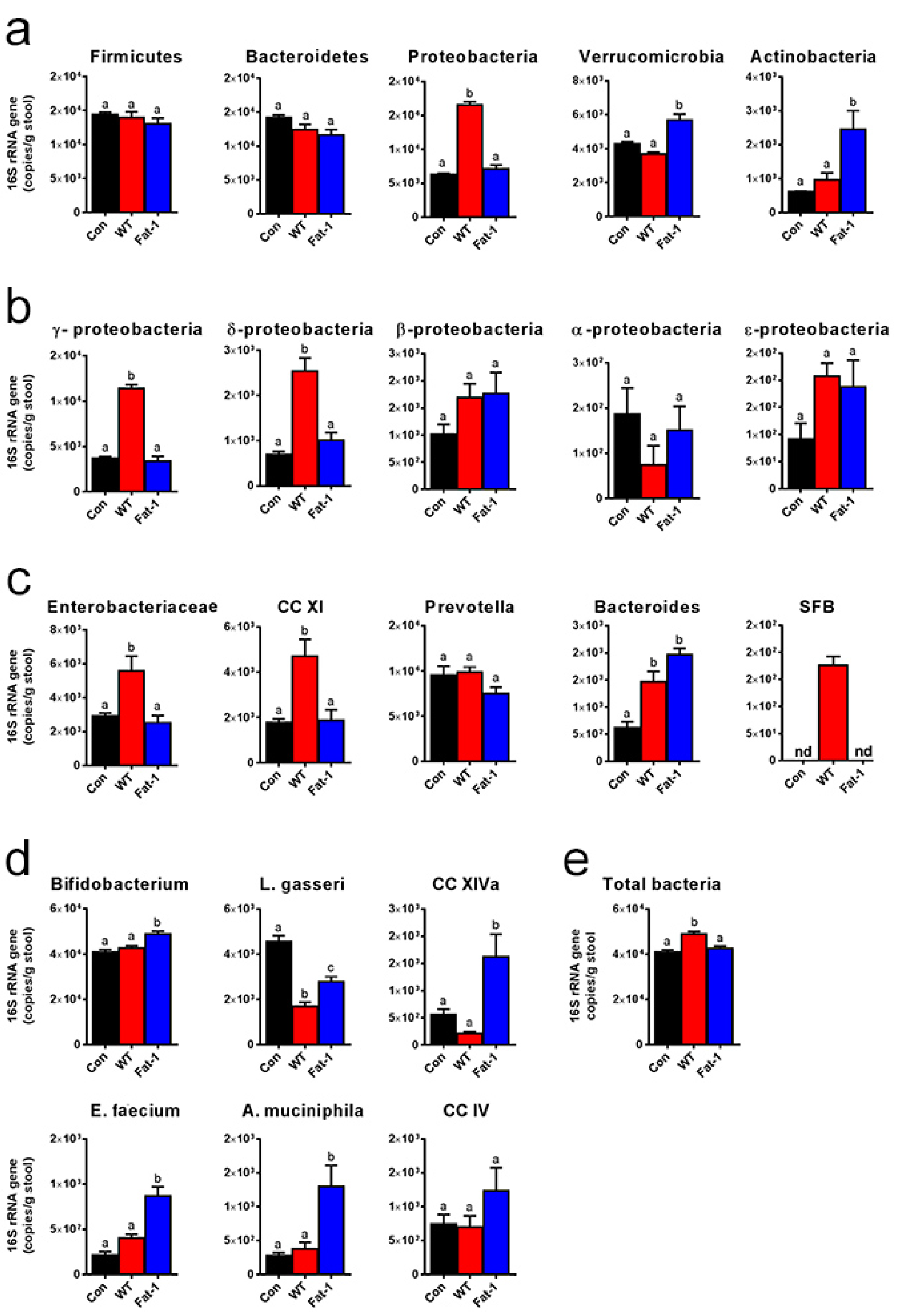

Supplementary Fig. S5. Opposing effects of tissue n-6 and n-3 PUFA on gut microbiota. Eight-month-old WT (n=10) and fat-1 (n=10) were maintained on the same high n-6 diet since weaning. A group of WT mice fed with a chow diet (n=10) was used as the control group. Stool samples from all groups of mice were subjected to quantification of microbiota by qPCR. **(a)** Copy numbers of five most dominant phylums in the stool between groups; **(b)** Major classes of LPS producing *Proteobacteria phylum* between groups; **(c)** Groups of LPS producing and/or pro-inflammatory bacteria at sub-phylum level; **(d)** Groups of LPS suppressing and/or anti-inflammatory bacteria at sub-phylum level; **(e)** Total bacteria**.** Data are expressed as mean ± SE. Data with different superscript letters are significantly different ($P < 0.05$) according to one-way ANOVA with Tukey Test.

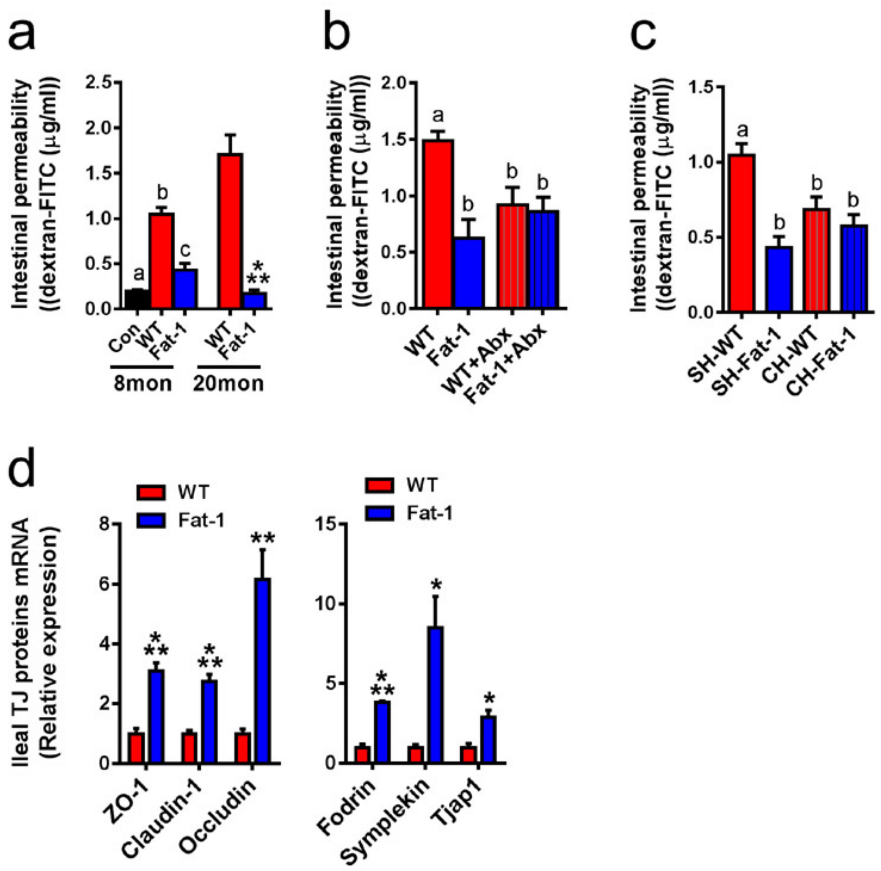

Supplementary Fig. S6. Effect of tissue n-6 and n-3 PUFA status on intestinal permeability and expression of tight junction proteins. (a) Difference in intestinal permeability between WT and fat-1 mice at the age of 8 months and 20 months; **(b)** Effect of antibiotics treatment on the difference in intestinal permeability between WT and fat-1 mice; **(c)** Effect of co-housing on the difference in intestinal permeability between WT and fat-1 mice; **(d)** Small intestinal mRNA expression of tight junction proteins (Zonulin-1, Claudin-1, Occludin, Fodrin, Symplekin and Tjap1) in the 20 month old WT and fat-1 mice. Data are expressed as mean ± SE. Data with different superscript letters are significantly different (P < 0.05) according to one-way ANOVA with Tukey Test, or * $P < 0.05$; ** $P < 0.01$; *** $P < 0.001$.

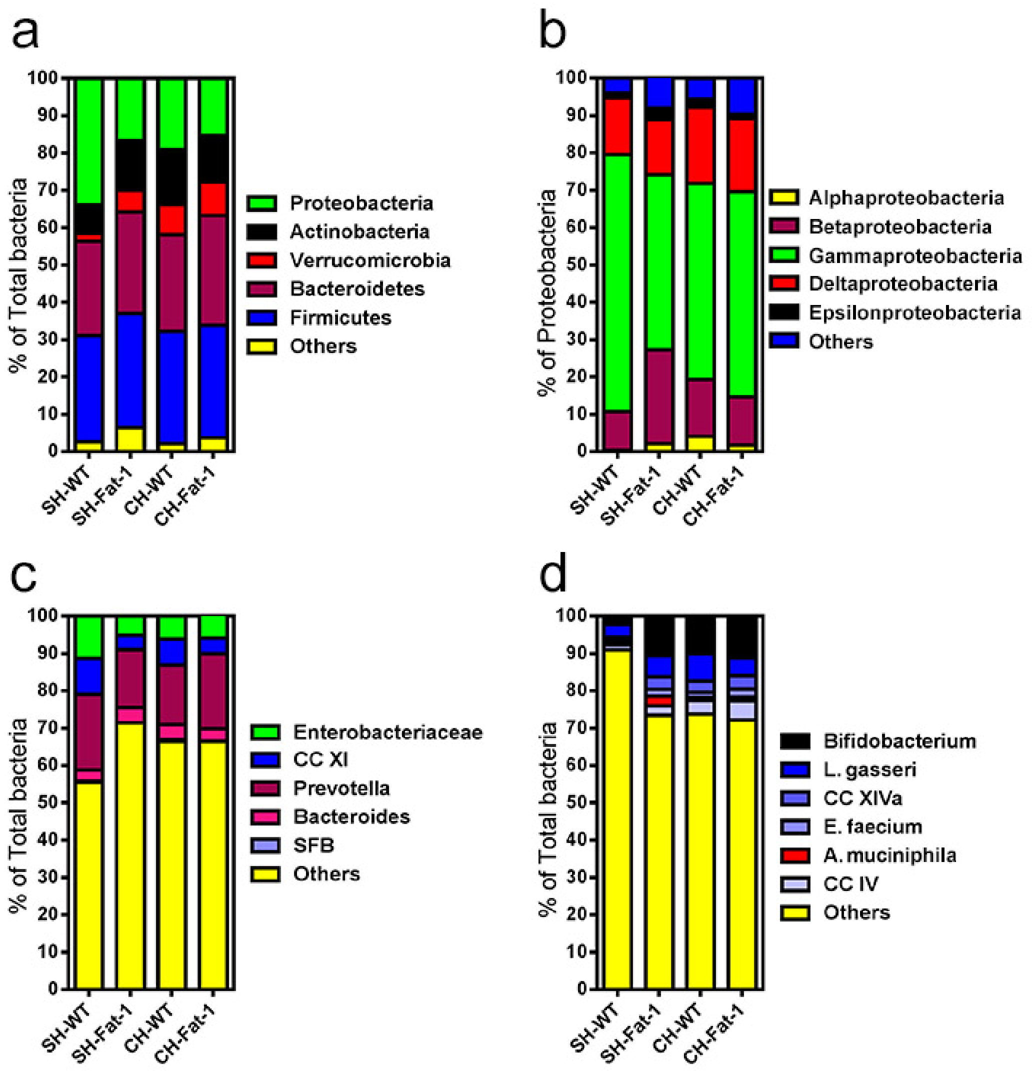

Supplementary Fig. S7. Co-housing of animals (WT+Fat-1) alleviates the differences between WT and fat-1 mice in LPS related gut microbiota profile (*Part 1: Relative abundance by percentage*). Stool samples were collected from separately housed (SH) mice and from 8-month co-housing (CH) mice and subjected to quantification of microbiota by qPCR. **(a)** Relative abundance for the 5 most dominant phylums in the stool between groups; **(b)** Relative abundance of the major classes of LPS-producing *Proteobacteria* phylum between groups; **(c)** Relative abundance of the major groups of LPS-producing and/or pro-inflammatory bacteria at the sub-phylum level; and **(d)** Relative abundance of major groups of LPS-suppressing and/or anti-inflammatory bacteria at the sub-phylum level.

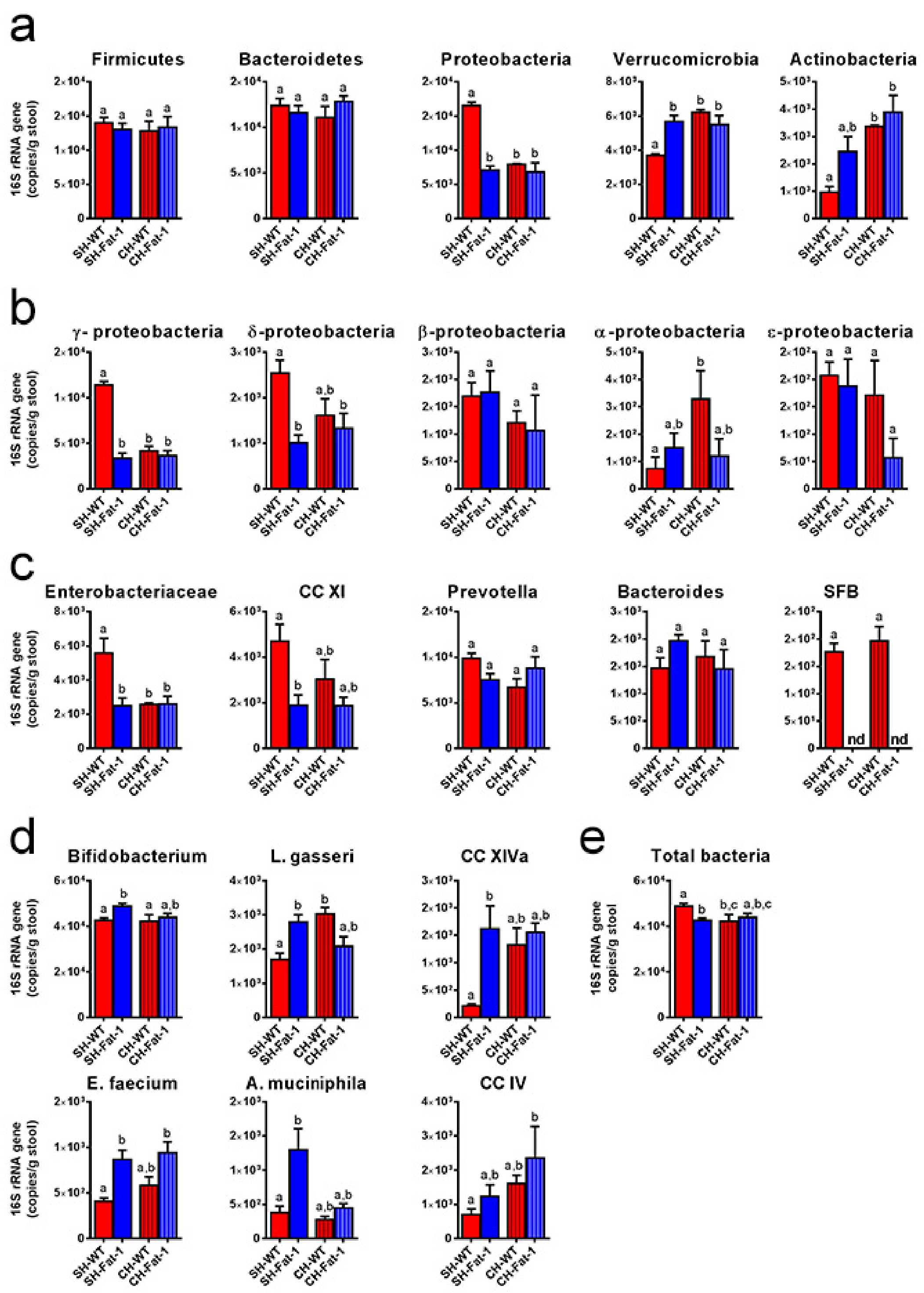

Supplementary Fig. S8. Co-housing of animals (WT+Fat-1) alleviates the differences between WT and fat-1 mice in LPS related gut microbiota profile (*Part 2: Absolute copy number*). Stool samples were collected from 8-month-old separately housed (SH) mice and co-housed (CH) mice, and subjected to quantification of microbiota by qPCR. **(a)** Copy numbers of five most dominant phylums in the stool between groups; **(b)** Major classes of LPS producing *Proteobacteria phylum* between groups; **(c)** Groups of LPS-producing and/or pro-inflammatory bacteria at sub-phylum level; **(d)** Groups of LPS-suppressing and/or anti-inflammatory bacteria at sub-phylum level; **(e)** Total bacteria**.** Data are expressed as mean ± SE. Data with different superscript letters are significantly different ($P < 0.05$) according to one-way ANOVA with Tukey Test.

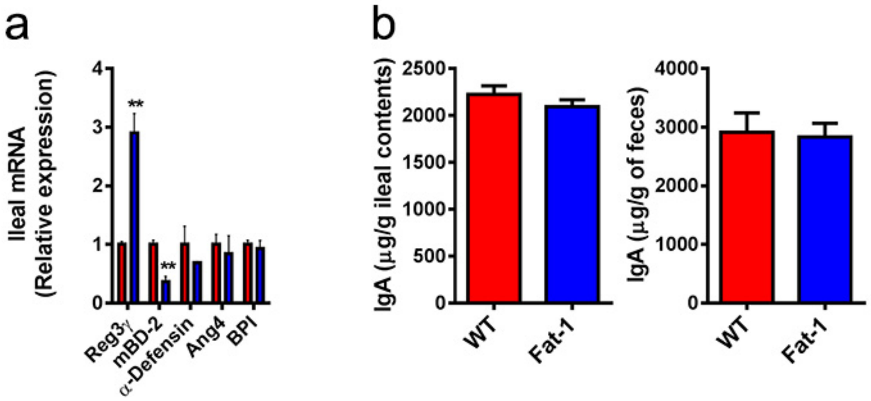

Supplementary Fig. S9. Separately housed 20 month old male WT (n=4) and fat-1 (n=5) were maintained on the same high n-6 PUFA diet since weaning. Ileal tissue and stool were collected from the mice and subjected to analysis for the levels of antimicrobial peptides, including Reg3γ, mBD-2, α-defensin, Ang4 and BPI **(a)** and IgA **(b)**. Data are expressed as mean ± SE. Significance was determined by unpaired two-tailed student T-test. **P* < 0.05; ** *P* < 0.01; *** *P* < 0.001.

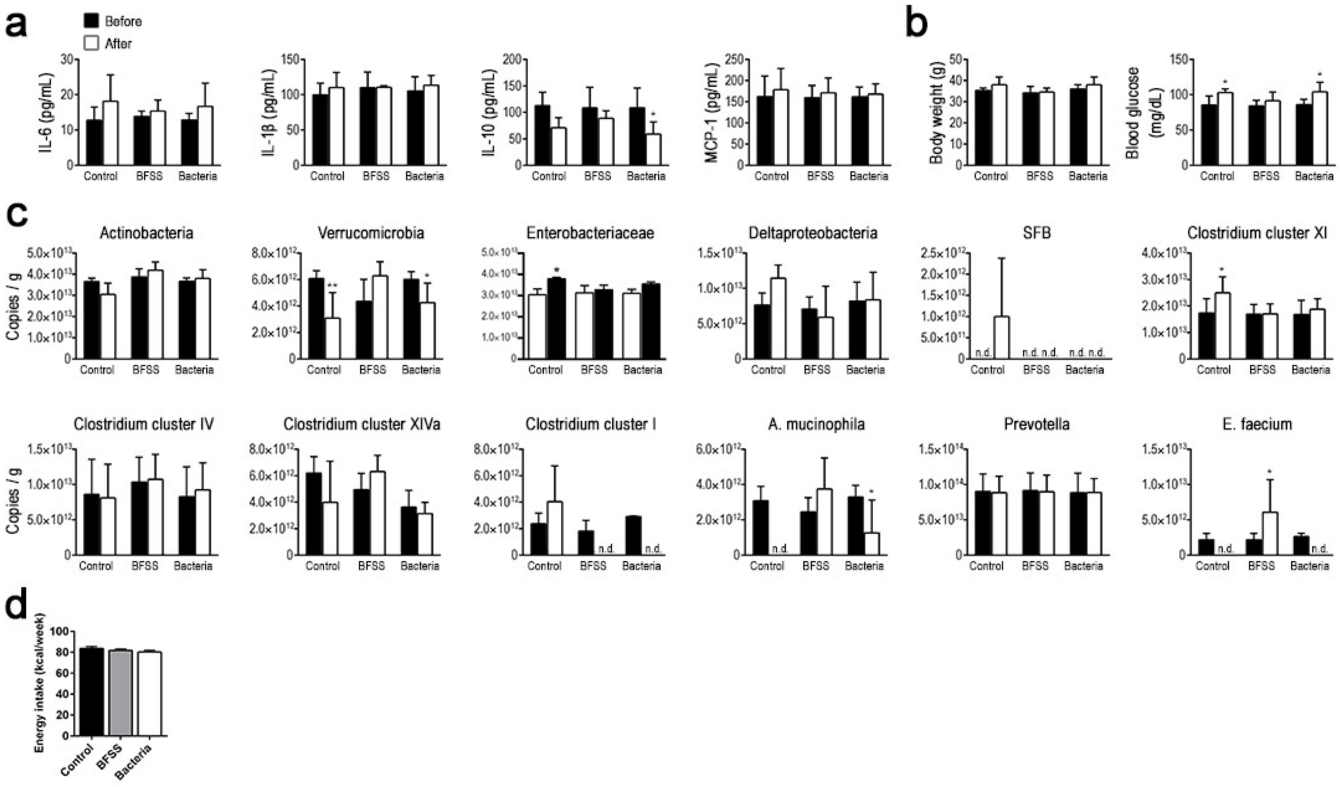

Supplementary Fig. S10. Transfer of fecal components from fat-1 to WT mice affects gut microbiota composition and parameters of inflammation. Fresh feces collected from fat-1 mice were separated into bacteria free stool supernatant (BFSS) and bacterial pellet fractions. The fecal BFSS and bacterial pellets from fat-1 mice were transferred by daily gavage or drinking water into WT mice that were simultaneously given a high n-6 diet. Two months after the treatments, the animals were subjected to analysis for changes in **(a)** markers of inflammation; **(b)** metabolic parameters; and **(c)** endotoxemia-related bacterial groups in the stool. **(d)** Energy intake. Data are expressed as mean ± SE. Significance was determined by paired T-test. *, $P < 0.05$; **, $P < 0.01$; nd, not detectable.

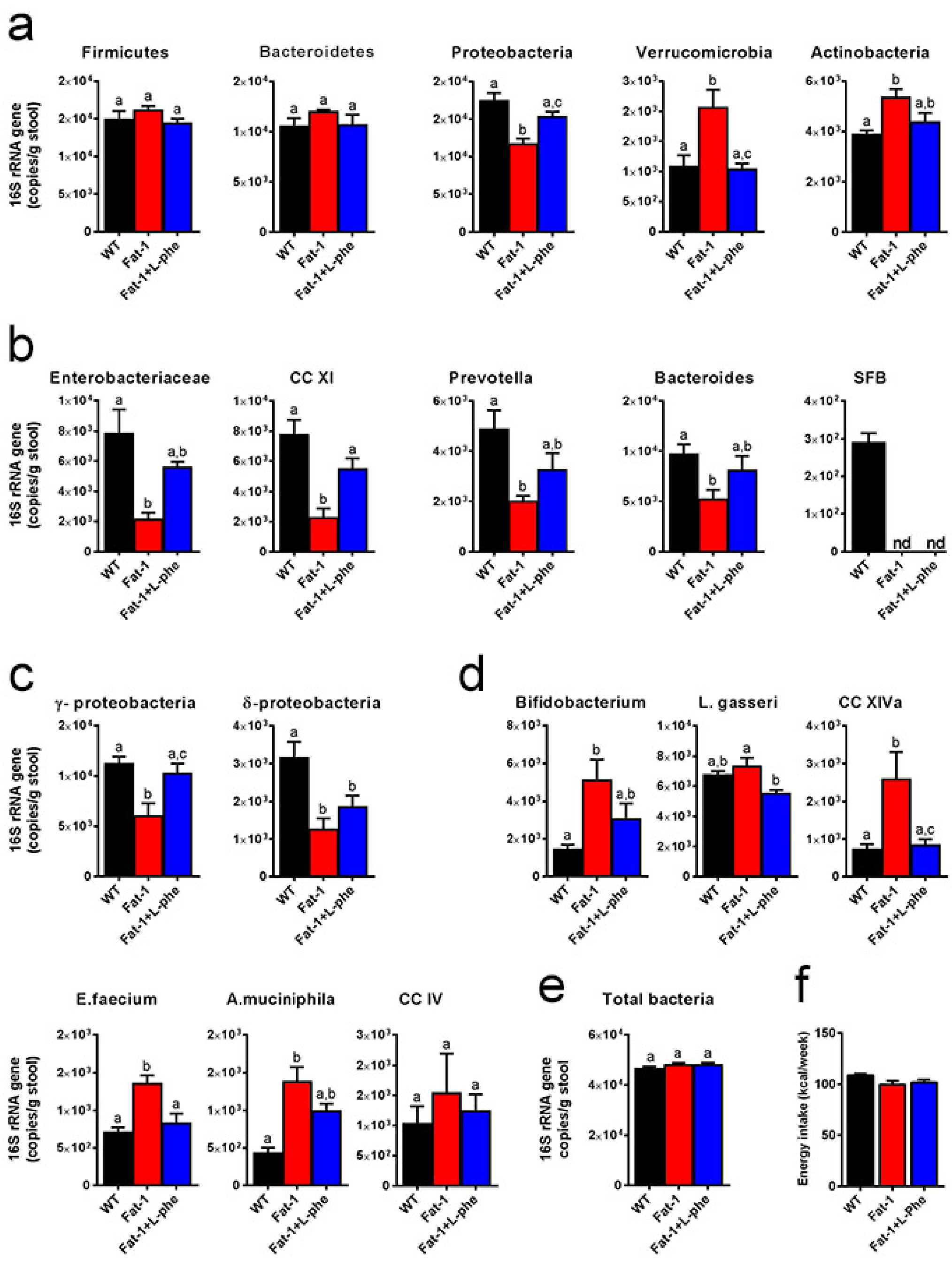

Supplementary Fig. S11. Inhibition of IAP in fat-1 mice was performed by adding an IAP-specific inhibitor (10Mm L-phenylalanine) to the drinking water of a subgroup of fat-1 mice for two months. The treated fat-1 mice (n=5) together with untreated fat-1 (n=5) and WT mice (n=5) were subjected to analysis for differences in the LPS related gut microbiota. **(a)** Copy numbers of the five most dominant phylums in the stool between groups; **(b)** Groups of LPS-producing and/or pro-inflammatory bacteria at sub-phylum level; **(c)** Major classes of LPS-producing *Proteobacteria* phylum between groups; **(d)** Major LPS-reducing bacteria; **(e)** Total bacteria; **(f)** Energy intake. Data are expressed as mean ± SE. Data with different superscript letters are significantly different ($P < 0.05$) according to one-way ANOVA with Tukey Test.

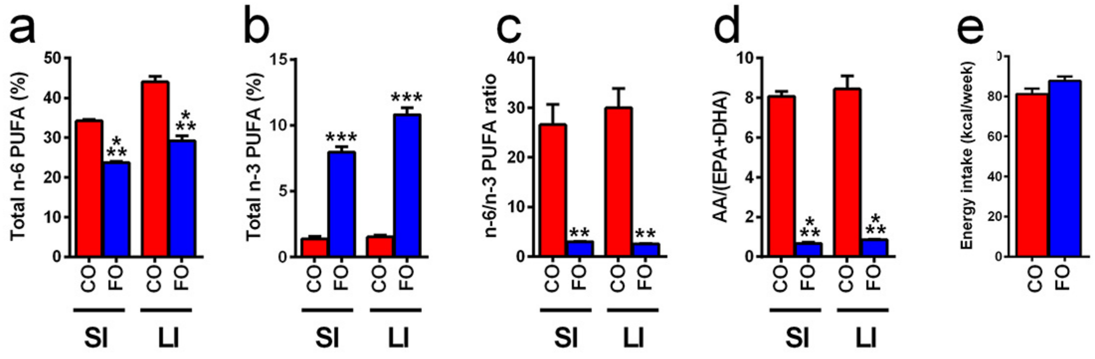

Supplementary Fig. S12. Alteration fatty acid profiles of the small (SI) and large (LI) intestinal tissues by fish oil supplementation. Twenty-month-old WT mice previously received a high n-6 PUFA (10% corn oil) diet were supplemented with n-3 PUFA (5% corn oil+5% fish oil) for 2 months. After sacrificing, small (SI) and large (LI) intestinal tissues were subjected to fatty acid analysis by gas chromatography. **(a)** Percentages of total n-6 PUFA; **(b)** Percentages of total n-3 PUFA; **(c)** Ratios of tissue n-6/n-3 PUFA; **(d)** AA/(EPA+DHA); **(e)** Energy intake. Values are expressed as mean ± SE. Significance was determined by unpaired two-tailed student T-test. (*$P < 0.05$, **$P < 0.01$, ***$P < 0.001$).

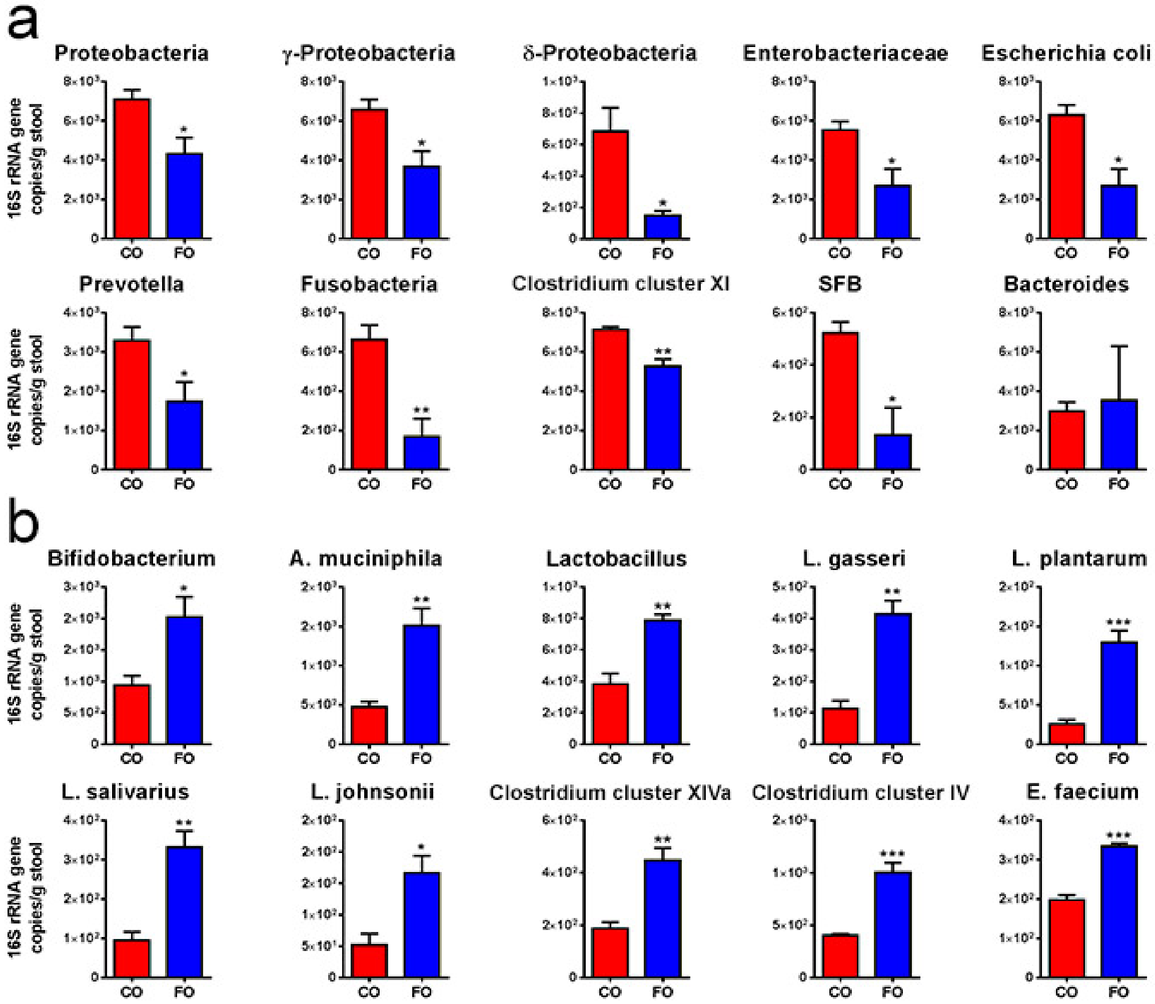

Supplementary Fig. S13. Elevating tissue n-3 PUFA status by fish oil supplementation alters gut bacterial profile. Twenty-month-old WT mice previously maintained on a high n-6 PUFA (10% corn oil) diet were supplemented with n-3 PUFA (5% corn oil+5% fish oil) for 2 months. Quantification of stool bacterial 16s rRNA copy numbers was determined by qPCR. **(a)** LPS-producing and/or pro-inflammatory bacteria; **(b)** LPS-reducing and/or anti-inflammatory bacteria. Values are expressed as mean ± SE. Significance was determined by unpaired two-tailed student T-test. (*$P < 0.05$, **$P < 0.01$, ***$P < 0.001$). CO, corn oil; FO, fish oil.

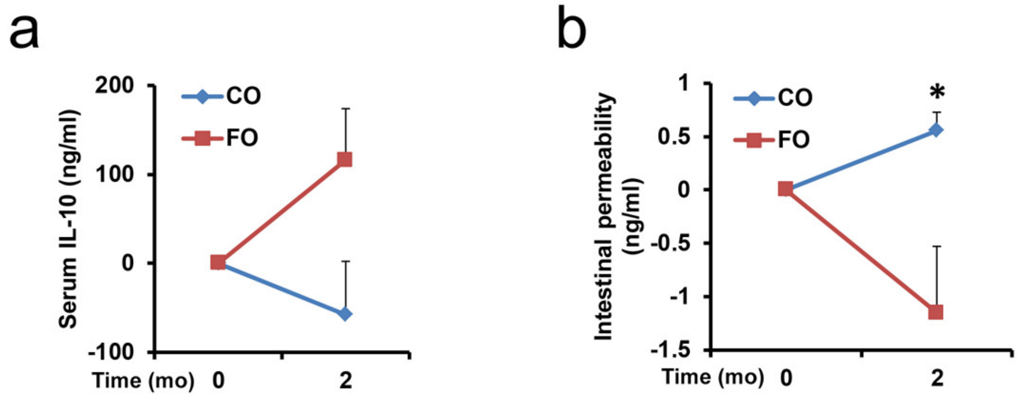

Supplementary Figure S14. Change in serum IL-10 and intestinal permeability by fish oil supplementation. Twenty-month-old WT mice previously maintained on a high n-6 PUFA (10% corn oil) diet were supplemented with n-3 PUFA (5% corn oil+5% fish oil) for 2 months. Serum was subjected to analysis of change in serum IL-10 **(a)** and intestinal permeability **(b)**. Data are expressed as mean ± SE. Significance was determined by unpaired two tailed student T-test *$P < 0.05$; **$P < 0.01$; ***$P < 0.001$.

Supplementary Table S1: Targeted LPS and inflammation-related gut microbiota.

Taxonomic rank	Target bacteria	LPS producing	LPS suppressing	Pro-inflammatory	Anti-inflammatory	Related to MetS/Inflammation	Reference
Phylum	PROTEOBACTERIA	Yes		Yes		Yes	(14, 16-18)
Class	α-proteobacteria						
Class	β-proteobacteria						
Class	γ-proteobacteria						
Class	δ-proteobacteria						
Class	ϵ-proteobacteria						
Family	Enterobacteriacea						
Genus	Escherichia coli						
Phylum	ACTINOBACTERIA		Yes		Yes	Yes	(19, 20)
Genus	Bifidobacterium						
Phylum	VERRUCOMICROBIA		Yes		Yes	Yes	(21, 22)
Species	Akkermansia mucinophilia						
Phylum	FUSOBACTERIA	Yes				Yes	(23, 24)
Phylum	BACTERIODETES	Yes				Yes	(25)
Genus	Bacteroides	Yes				Yes	(14)
Genus	Prevotella	Yes			Yes		(26)
Phylum	FIRMICUTES					Yes	(25)
Genus	Lactobacillus				Yes	Yes	(27-29)
Family	Clostridium cluster XIVa						
Family	Clostridium cluster IV						
Family	Clostridium cluster I						
Family	Roseburia						
Genus	Clostridium cluster XI			Yes		Yes	(10)
Species	Segmented Filamentous Bacteria			Yes	Yes	Yes	(14)
Species	Faecalibacterium prausnitzii					Yes	(30)
Species	Enterococcus faecium		Yes			Yes	(31)
Species	Lactobacillus gasseri		Yes		Yes	Yes	(32)
Species	Lactobacillus plantarum						
Species	Lactobacillus johnsonii		Yes		Yes	Yes	(33, 34)
Species	Lactobacillus acidophilus		Yes		Yes	Yes	(34)
Species	Lactobacillus fermentum		Yes		Yes	Yes	(35)

Supplementary Table S2: Fatty acid composition of experimental diets.

%	Chow diet	High n-6 diet (10% corn oil)	High n-3 diet (5% corn oil + 5% fish oil)
C6:0			
C8:0	0.08	0.11	
C10:0	0.1	0.27	0.03
C12:0	1.9	0.7	0.2
C14:0			0.12
C14:1	0.18		0.02
C16:1	2.43	0.5	0.18
C17:0	0.24		0.15
C17:1	0.13		
C18:0	15.2	2.36	3.31
C18:1	13	28.2	13.9
C18:2	39.2	52.3	18
C18:3	4.07	1.1	0.91
C20:0			0.39
C20:1	0.39		1.44
C20:4	0.25		1.67
C20:5	1.49		31.7
C22:5	0.11		3.8
C22:6	0.84		18.8
SFA	37.9	18.2	9.37
MUFA	16.2	28.4	15.9
PUFA	45.9	53.4	74.8
n-6 PUFA	39.4	52.3	19.7
n-3 PUFA	6.51	1.1	55.2
n-6/n-3	6.05	47.6	0.36

Supplementary Table S3: Target organisms, reference bacterial genomic DNA, and oligonucleotide primers.

Taxonomic rank	Target organism	Reference bacterial genomic DNA@	**Sequence 5' - 3'**
Kingdom	All bacteria	NR-2655- Escherichia coli K-12, Strain MG1655	F. ACTCCTACGGGAGGCAGCAGT<br>R. ATTACCGCGGCTGCTGGC
Phylum	Firmicutes (Gram positive)	HM-102D-Lactobacillus reuteri, Strain CF48-3A	F. GGAGYATGTGGTTTAATTCGAAGCA<br>R. AGCTGACGACAACCATGCAC
	Genus<br>Lactobacillus	HM-102D-Lactobacillus reuteri, Strain CF48-3A	F. AGCAGTAGGGAATCTTCCA<br>R. CAC CGC TAC ACA TGG AG
	Family<br>Clostridium cluster XI	NR-13592-Clostridium difficile, Isolate 1	F. TGACGGTACYYNRKGAGGAAGCC<br>R. ACTACGGTTRAGCCGTAGCCTTT
	Species<br>Roseburia	HM-84D-Clostridiales (Deposited as Hespelliasp., Strain 3_1_39B/D5) Clostridiales bacterium	F. TACTGCATTGGAAACTGTCG<br>R. CGGCACCGAAGAGCAAT
	Family<br>Clostridium cluster XIVa	HM-480D-Lachnospiraceae sp., Strain ACC2	F. GCGGTRCGGCAAGTCTGA<br>R. CCTCCGACACTCTAGTMCGAC
	Family<br>Clostridium cluster IV	HM-84D-Clostridiales (Deposited as Hespelliasp., Strain 3_1_39B/D5) Clostridiales bacterium	F. GCACAAGCAGTGGAGT<br>R. CTTCCTCCGTTTTGTCAA
	Family<br>Clostridium cluster I	HM-310D-Clostridium perfringens, Strain WAL-14572	F. AAAGGAAGATTAATACCGCATA<br>R. TTCTTCCTAATCTCTACGCA
	Species<br>Segmented Filamentous Bacteria	HM-36D-Clostridium sp., Strain 7_2_43FAA	F. GACGCTGAGGCATGAGAGCA<br>R. GACGGCACGGATTGTTATTC
	Species<br>Faecalibacterium prausnitzii	HM-473 Faecalibacterium prausnitzii KLE1255	F. CCATGAATTGCCTTCAAAACTGTT<br>R. GAGCCTCAGCGTCAGTTGGT
	Species<br>Enterococcus faecium	HM-204D-Enterococcus faecium, Strain TX1330	F. TGCTCCACCGGAAAAAGA<br>R. CACCAACTAGCTAATGCA
	Species<br>Lactobacillus gasseri	HM-104D-Lactobacillus gasseri, Strain JV-V03	F. TGGAAACAGRTGCTAATACCGR.<br>CAGTTACTACCTCTATCTTTCTTCACTAC
	Species<br>Lactobacillus johnsonii	HM-643 Lactobacillus johnsonii 135-1-CHN	F. CAAAAACCAACTTTCTATGTG<br>R. TTAATAGTGGCTACCCATA
	Species<br>Lactobacillus plantarum	HM-261 Lactobacillus plantarum KCA-1	F. CTCTGGTATTGATTGGTGCTTGCAT<br>R. GTTCGCCACTCACTCAAATGTAAA
	Species<br>Lactobacillus salivarius	HM-228 Lactobacillus sp. 7_1_47FAA	F. CGAAACTTTCTTACACCGAATGC<br>R. GTCCATTGTGGAAGATTCCC
	Species<br>Lactobacillus acidophilus	HM-228 Lactobacillus sp. 7_1_47FAA	F. GCAGATCGCATGATCAGCTTATA<br>R. TCAGTCTCTCAACTCGGCTATG
	Species<br>Lactobacillus fermentum	HM-228 Lactobacillus sp. 7_1_47FAA	F. AACCGAGAACACCGCGTTAT<br>R.ACTTAACCTTACTGATCGTAGATCAGTCA
Phylum	Bacteroidetes (Gram negative)	HM-23D-Bacteroides sp., Strain 1_1_6	F. GGARCATGTGGTTTAATTCGATGAT<br>R. AGCTGACGACAACCATGCAG
	Genus<br>Bacteroides	HM-23D-Bacteroides sp., Strain 1_1_6	F. AAGGTCCCCCACATTGG<br>R. GAGCCGCAAACTTTCACAA
	Genus<br>Prevotella	HM-80D-Prevotella melaninogenica, Strain D18	F. GGTTCTGAGAGGAAGGTCCCC<br>R. TCCTGCACGCTACTTGGCTG
Phylum	Proteobacteria (Gram negative)	NR-2655-Escherichia coli K-12, Strain MG1655	F. CATGACGTTACCCGCAGAAGAAG<br>R. CTCTACGAGACTCAAGCTTGC
	Class<br>α-Proteobacteria	NR-10486-Rickettsia sibirica, Strain 246	F. CIAGTGTAGAGGTGAAATT<br>R. CCCCGTCAATTCCTTTGAGTT
	Class<br>β-Proteobacteria	NR-2655-Escherichia coli K-12, Strain MG1655	F. GGGGAATTTTGGACAATGGG<br>R. ACGCATTTCACTGCTACACG
	Class<br>γ-Proteobacteria	NR-2655-Escherichia coli K-12, Strain MG1655	F. CMATRCCGCGTGTRTGAA<br>R. ACTCCCCAGGCGGTCDACTTA
	Class<br>δ-Proteobacteria	HM-66D-Desulfovibrio sp., Strain 3_1_syn3	F. GGTGTAGGAGTGAARTCCGT<br>R. TACGTGTGTAGCCCTRGRC
	Class<br>€-Proteobacteria	NR-4119-Campylobacter jejuni, Strain UA466	F. TGGTGTAGGGGTAAAATCCG<br>R. AGGTAAGGTTCTTCGYGTATC
	Family<br>Enterobacteriaceae	NR-2655-Escherichia coli K-12, Strain MG1655	F. GTGCCAGCAGCCGCGGTAA<br>R. GCCTCAAGGGCACAACCTCCAAG
	Genus<br>Escherichia coli	NR-2655-Escherichia coli K-12, Strain MG1655	F. CATGCCGCGTGTATGAAGAA<br>R. CGGGTAACGTCAATGAGCAAA
Phylum	Actinobacteria (Gram positive)	HM-30D-Bifidobacterium sp., Strain 12_1_47BFAA	F. CGCGGCCTATCAGCTTGTTG<br>R. CCGTACTCCCCAGGCGGGG
	Genus<br>Bifidobacterium	HM-30D-Bifidobacterium sp., Strain 12_1_47BFAA	F. CGGGTGAGTAATGCGTGACC<br>R. TGATAGGACGCGACCCCA
Phylum	Verrucomicrobia (Gram negative)	Akkermansia muciniphila Derrien et al (ATCC® BAA-835D-5™)#	F. GAATTCTCGGTGTAGCA<br>R. GGCATTGTAGTACGTGTGCA
	Species<br>Akkermansia muciniphila	Akkermansia muciniphila Derrien *et al.* (ATCC® BAA-835D-5™)#	F. CAGCACGTGAAGGTGGGGAC<br>R. CCTTGCGGTTGGCTTCAGAT
Phylum	Fusobacteria (Gram negative)	HM-260D-Fusobacterium nucleatum subsp.polymorphum, Strain F0401	F. GATCCAGCAATTCTGTGTGC<br>R. CGAATTTCACCTCTACACTTGT

@ - Reagent was obtained through BEI Resources, NIAID, NIH as part of the Human Microbiome Project.

# - Reagent was ordered from American Type Culture Collection (ATCC), VA, USA.

Supplementary Table S4: Primer sets used for real-time quantitative PCR.

**Gene**	**Forward primer** (Sequence 5ʹ- 3ʹ)	**Reverse primer** (Sequence 5ʹ- 3ʹ)
ZO-1	TCTACGAGGGACTGTGGATG	TCAGATTCAGCAAGGAGTCG
Claudin-1	TCTACGAGGGACTGTGGATG	TCAGATTCAGCAAGGAGTCG
Occludin	ACCCGAAGAAAGATGGATCG	CATAGTCAGATGGGGGTGGA
Fodrin	CGCATCTTTTTCCTCAGCAG	CCAGGACTTGCTGTCGTCTC
Symplekin	TGAGGGCTGAGAAGGCTGTA	CAGCACCTCTGCCTTGAATC
Tjap1 (7H6)	CTCCAGAGCACCGAGAGCTA	GCGTTTGCGAAGTTCTTCAT
TLR4	CGCTTTCACCTCTGCCTTCACTACAG	ACACTACCACAATAACCTTCCGGCTC
AKP3	ACATTGCTACACAACTCATCTCC	TCCTGCCATCCAATCTGGTTC
AKP6	AGGATCCATCTGTCCTTTGGT	CAGCTGCCTTCTTGTTCC
Reg3γ	TTCCTGTCCTCCATGATCAAA	CATCCACCTCTGTTGGGTTC
mBD-2	TCTCTGCTCTCTGCTGCTGATATGC	AGGACAAATGGCTCTGACACAGTACC
Ang4	GCTGGGTCTGGTTGTGATTCC	AGGCGAGGTTAGCTTTCTTTCC
Global α-defensins	GGTGATCATCAGACCCCAGCATCAGT	AAGAGACTAAAACTGAGGAGCAGC
BPI	AGCAGGGAGTGGTTGAGT	GATGGTGGTGATGTGGC-
LBP	GGGTCTGCAGAGAGAGCTGTACAA	TAGTTAAGGAATGCCTGGAAC
CD14	ACATCTTGAACCTCCGCAAC	AGGGTTCCTATCCAGCCTGT
NLRP3	AGCCTTCCAGGATCCTCTTC	CTTGGGCAGCAGTTTCTTTC
ASC	GAAGCTGCTGACAGTGCAAC	GCCACAGCTCCAGACTCTTC
Caspase-1	AGATGGCACATTTCCAGGAC	GATCCTCCAGCAGCAACTTC
Pannexin-1	CTCGGACTTCTTGCTGAAGG	TACAGCAGCCCAGCAGTATG
β-actin	GAGAAGATCTGGCACCACACC	GCATACAGGGACAGCACAGC
	GATTACTGCTCTGGCTCCTAGC	GACTCATCGTACTCCTGCTTGC

Supplementary References

1. Kang JX, Wang J, Wu L, Kang ZB. Transgenic mice: fat-1 mice convert n-6 to n-3 fatty acids. *Nature* **427**, 504 (2004).
2. Reikvam DH, *et al.* Depletion of murine intestinal microbiota: effects on gut mucosa and epithelial gene expression. *PLoS One* **6**, e17996 (2011).
3. Bel S, *et al.* Reprogrammed and transmissible intestinal microbiota confer diminished susceptibility to induced colitis in TMF-/- mice. *Proc Natl Acad Sci USA* **111**, 4964-4969 (2014).
4. Prizont R, Konigsberg N. Identification of bacterial glycosidases in rat cecal contents. *Dig Dis Sci* 26, 773-777 (1981).
5. Moss AK, *et al.* Intestinal alkaline phosphatase inhibits the proinflammatory nucleotide uridine diphosphate. *Am J Physiol Gastrointest Liver Physiol* **304**, G597-604 (2013).
6. Peng J, *et al.* Long term effect of gut microbiota transfer on diabetes development. *J Autoimmun* **53**, 85-94 (2014).
7. Campbell EL, *et al.* Resolvin E1-induced intestinal alkaline phosphatase promotes resolution of inflammation through LPS detoxification. *Proc Natl Acad Sci USA* **107**, 14298-14303 (2010).
8. Kaliannan K, *et al.* Intestinal alkaline phosphatase prevents metabolic syndrome in mice. *Proc Natl Acad Sci USA* **110**, 7003-7008 (2013).
9. Alam SN, *et al.* Intestinal alkaline phosphatase prevents antibiotic-induced susceptibility to enteric pathogens. *Ann Surg* **259**, 715-722 (2014).
10. Cowan TE, *et al.* Chronic coffee consumption in the diet-induced obese rat: impact on gut microbiota and serum metabolomics. *J Nutr Biochem* **25**, 489-495 (2014).
11. Louie TJ, *et al.* Fidaxomicin preserves the intestinal microbiome during and after treatment of Clostridium difficile infection (CDI) and reduces both toxin reexpression and recurrence of CDI. *Clin Infect Dis* **55 Suppl 2**, S132-142 (2012).
12. Kim KJ, Lee OH, Lee BY. Low-molecular-weight fucoidan regulates myogenic differentiation through the mitogen-activated protein kinase pathway in C2C12 cells. *Br J Nutr* **106**, 1836-1844 (2011).
13. Kang JX, Wang J. A simplified method for analysis of polyunsaturated fatty acids. *BMC Biochem* **6**, 5 (2005).
14. Ghosh S, *et al.* Fish oil attenuates omega-6 polyunsaturated fatty acid-induced dysbiosis and infectious colitis but impairs LPS dephosphorylation activity causing sepsis. *PLoS One* **8**, e55468 (2013).
15. Mohammadi A, Oshaghi EA. Effect of garlic on lipid profile and expression of LXR alpha in intestine and liver of hypercholesterolemic mice. *J Diabetes Metab Disord* **13**, 20 (2014).
16. de La Serre CB, *et al.* Propensity to high-fat diet-induced obesity in rats is associated with changes in the gut microbiota and gut inflammation. *Am J Physiol Gastrointest Liver Physiol* **299**, G440-448 (2010).
17. Zhang C, *et al.* Interactions between gut microbiota, host genetics and diet relevant to development of metabolic syndromes in mice. *ISME J* **4**, 232-241 (2010).
18. Festi D, *et al.* Gut microbiota and metabolic syndrome. *World J Gastroenterol* **20**, 16079-16094 (2006).
19. Riedel CU, *et al.* Anti-inflammatory effects of bifidobacteria by inhibition of LPS-induced NF-kappaB activation. *World J Gastroenterol* **12**, 3729-3735 (2006).
20. Cani PD, *et al.* Selective increases of bifidobacteria in gut microflora improve high-fat-diet-induced diabetes in mice through a mechanism associated with endotoxaemia. *Diabetologia* **50**, 2374-2383 (2007).
21. Everard A, *et al.* Cross-talk between Akkermansia muciniphila and intestinal epithelium controls diet-induced obesity. *Proc Natl Acad Sci USA* **110**, 9066-9071 (2013).

22. Anhê FF, *et al.* A polyphenol-rich cranberry extract protects from diet-induced obesity, insulin resistance and intestinal inflammation in association with increased Akkermansia spp. population in the gut microbiota of mice. *Gut* doi:10.1136/gutjnl-2014-307142 (2014).
23. Chen Y, *et al.* Characterization of fecal microbial communities in patients with liver cirrhosis. *Hepatology* **54**, 562-572 (2011).
24. Yu LX, *et al.* Endotoxin accumulation prevents carcinogen-induced apoptosis and promotes liver tumorigenesis in rodents. *Hepatology* **52**, 1322-1333 (2010).
25. Turnbaugh PJ, *et al.* An obesity-associated gut microbiome with increased capacity for energy harvest. *Nature* **444**, 1027-1031 (2006).
26. Demon D, *et al.* Caspase-11 is expressed in the colonic mucosa and protects against dextran sodium sulfate-induced colitis. *Mucosal Immunol* **7**, 1480-1491 (2014).
27. Atarashi K, *et al.* Induction of colonic regulatory T cells by indigenous Clostridium species. *Science* 331, 337-341 (2011).
28. Bajaj JS, *et al.* Randomised clinical trial: Lactobacillus GG modulates gut microbiome, metabolome and endotoxemia in patients with cirrhosis. *Aliment Pharmacol Ther* **39**, 1113-1125 (2014).
29. Naito E, *et al.* Beneficial effect of oral administration of Lactobacillus casei strain Shirota on insulin resistance in diet-induced obesity mice. *J Appl Microbiol* **110**, 650-657 (2011).
30. Sokol H, *et al.* Faecalibacterium prausnitzii is an anti-inflammatory commensal bacterium identified by gut microbiota analysis of Crohn disease patients. *Proc Natl Acad Sci USA* **105**, 16731-16736 (2008).
31. Cao GT, *et al.* Effects of a probiotic, Enterococcus faecium, on growth performance, intestinal morphology, immune response, and cecal microflora in broiler chickens challenged with Escherichia coli K88. *Poult Sci* **92**, 2949-2955 (2013).
32. Million M, *et al.* Comparative meta-analysis of the effect of Lactobacillus species on weight gain in humans and animals. *Microb Pathog* **53**, 100-108 (2012).
33. Miyoshi M, Ogawa A, Higurashi S, Kadooka Y. Anti-obesity effect of Lactobacillus gasseri SBT2055 accompanied by inhibition of pro-inflammatory gene expression in the visceral adipose tissue in diet-induced obese mice. *Eur J Nutr* **53**, 599-606 (2014).
34. Xin J, *et al.* Preventing non-alcoholic fatty liver disease through Lactobacillus johnsonii BS15 by attenuating inflammation and mitochondrial injury and improving gut environment in obese mice. *Appl Microbio Biotechnol* **98**, 6817-6829 (2014).
35. Lee SJ, *et al.* The effects of co-administration of probiotics with herbal medicine on obesity, metabolic endotoxemia and dysbiosis: A randomized double-blind controlled clinical trial. *Clin Nutr* **33**, 973-981 (2014).
